# Topical Application of Adult Cecal Contents to Eggs Transplants Spore-Forming Microbiota but Not Other Members of the Microbiota to Chicks

**DOI:** 10.1128/AEM.02387-19

**Published:** 2020-02-18

**Authors:** Peter Richards-Rios, Gail Leeming, Jo Fothergill, Marion Bernardeau, Paul Wigley

**Affiliations:** a Institute of Infection and Global Health, University of Liverpool, Liverpool, United Kingdom; b Department of Veterinary Pathology, Infection and Public Health, Institute of Veterinary Science, University of Liverpool; c DuPont Industrial Biosciences, Genencor International BV, Leiden, The Netherlands; Rutgers, The State University of New Jersey

**Keywords:** broiler, cecum, chicken, egg, microbiome, transplant

## Abstract

The intestinal microbiota plays an essential role in the metabolism and immune competence of chickens from the first day after hatching. In modern production systems, chicks are isolated from adult chickens, instead hatching in a clean environment. As a result, chicks are colonized by environmental bacteria, including potential pathogens. There is a need to investigate methods by which chicks can be exposed to a more appropriate microbial community at hatching. Such methods must be easy to apply in a hatchery and produce consistent results. The development of the intestinal microbiota of chicks hatched from eggs sprayed with dilute adult cecal content during incubation was observed at 0, 3, 7, and 14 days posthatching (dph) across two experiments. High-throughput Illumina sequencing was performed for the V4 hypervariable region of the 16S rRNA gene. A topical treatment of dilute adult cecal content was sufficient to transplant spore-forming bacteria such as *Lachnospiraceae* and *Ruminococcaceae*. However, this treatment was not able to transplant other taxa that are considered to be core elements of the chicken cecal microbiota, such as *Bacteroidaceae*, *Lactobacillaceae*, *Bifidobacteriaceae*, and *Burkholderiaceae*. The topical treatment significantly altered the microbiota of chicks immediately posthatching and accelerated the normal development of the microbiota with earlier colonization by *Ruminococcaceae* in the cecum and “Candidatus *Arthromitus*” in the ileum. The effect of the treatment on the cecal microbiota was maximal at 3 dph but diminished over time.

**IMPORTANCE** Over the last 60 years poultry production has intensified in response to increased demand for meat. In modern systems, chicks hatch without contacting chickens and their gut bacteria. Consequently, they are colonized by environmental bacteria that may cause disease. The normal bacteria that live in the gut, or intestinal microbiota, play an important role in the development of the immune system. Therefore, it is essential to find easy ways to expose chicks to the more appropriate bacteria at hatching. This experiment investigated whether spraying eggs with adult cecal contents was sufficient to transfer an adult microbiota to chicks. Our findings show that spore-forming bacteria were transplanted, but other members of the microbiota were not. In this respect, the spray application was partially successful, but the timing of the spray needs to be modified to ensure that more bacteria are transferred.

## Abstract (precis)

Over the last 60 years poultry production has intensified in response to increased demand for meat. In modern systems, chicks hatch without contacting chickens and their gut bacteria. Consequently, they are colonized by environmental bacteria that may cause disease. The normal bacteria that live in the gut, or intestinal microbiota, play an important role in the development of the immune system. Therefore, it is essential to find easy ways to expose chicks to the more appropriate bacteria at hatching. This experiment investigated whether spraying eggs with adult cecal contents was sufficient to transfer an adult microbiota to chicks. Our findings show that spore-forming bacteria were transplanted, but other members of the microbiota were not. In this respect, the spray application was partially successful, but the timing of the spray needs to be modified to ensure that more bacteria are transferred.

## INTRODUCTION

Between 1961 and 2001, the global average annual meat consumption per capita nearly doubled from 23.1 kg to 42.20 kg (1). Much of this increase has been provided by a growing poultry industry, which has intensified and industrialized to meet demand. The broiler industry in the United Kingdom produced approximately 82 million broilers per month in 2018 (2). The industrialization of poultry production has led to the separation of mature adults, eggs, and immature chickens at independent sites. Breeder flocks, constituting the genetic elite of the national chicken population, are kept exclusively to provide fertile eggs. Eggs are transported to hatcheries, which may be on the same site, where they are incubated in batches until hatching. After hatching, chicks are sold as “day-old chicks” to finishers, where they remain until slaughter at around 42 days old. This separation of chicks from maternal contact delays colonization of the gut by normal commensals (3). Instead, chicks are first colonized by environmental bacteria in hatcheries and during transport, which may include possible pathogens such as Clostridium perfringens or Escherichia coli (4–6). Poultry flocks are exquisitely sensitive to the presence of enteric pathogens (7, 8). While many can be controlled using vaccination and biosecurity, pathogens such as *Campylobacter* remain rife within the UK chicken population (9). Prophylactic use of antibiotics in the poultry industry was widely adopted as a growth promoter, with a secondary effect of facilitating enteric pathogen control and reducing production losses (10). However, the indiscriminate use of antibiotics has led to a rise in antimicrobial resistance. In order to combat this threat to public health, the European Union enacted a ban on the use of antibiotics as growth promoters in 2006 (11). As a result, this tool of the poultry industry must be replaced with alternatives.

Manipulation of the intestinal microbiota provides one such alternative. Many efforts to alter the microbiota have focused on the introduction of probiotics via feed or water to growing and adult chicks (12). However, a growing body of evidence suggests that the ability to influence microbiota composition decreases with age as a stable microbial community is established (13). Questions remain concerning the optimal timing and delivery mechanism for microbiota interventions. Until recently, the embryonic gut was thought to be sterile. With the advent of molecular techniques, this assumption of sterility has been challenged, with some evidence showing the presence of bacteria in the embryonic gut. Molecular techniques have been used to visualize and detect bacteria in embryonic chick tissue. For example, viable bacteria were detected in the cecal tissue from embryos at 18 and 20 days of incubation (d.i.) using fluorescence *in situ* hybridization (14). Bacterial DNA from *Enterobacteriaceae*, *Actinomycetales*, *Bifidobacteriales*, and *Lachnospiraceae* was detected using terminal restriction fragment length polymorphism (T-RFLP) of the entire gastrointestinal tract of chicken embryos (15), raising the possibility that *in ovo* microbial colonization occurs in proximal parts of the gastrointestinal tract as well as the cecum. However, skepticism about such results is not unwarranted, as low microbial biomass samples are known to be prone to contamination leading to false-positive results and inflated microbial diversity (16). The presence of bacteria within embryos and eggs would pose a question as to their origin. Vertical transmission is one possibility but is considered unlikely (17). Germ-free chicks can be derived by sterilizing the eggshell immediately after laying and rearing them in an isolator, indicating that vertical transmission would be an uncommon route of colonization for normal microbiota (18, 19). This suggests that the principal entry route for bacteria would be penetration of the eggshell and subsequent egg defenses. Most studies focus on the ability of *Salmonella* and other bacteria of public health importance to translocate from the eggshell to the embryo, although one study does demonstrate that other bacterial taxa are able to penetrate the eggshell (20). While these findings demonstrated that penetration of the eggshell is possible by certain bacterial taxa, it cannot be taken as evidence that microbes on the egg surface are able to traverse the albumen and successfully colonize the embryonic gut. An aim of this study was to detect bacteria within the embryonic gut and to resolve whether a selection of commensal bacteria applied to the egg surface during incubation would be detected in the embryonic gut.

This study also aimed to investigate the effect of a topical application of adult cecal content on the development of the chicken intestinal microbiota and identify which bacterial taxa can be transplanted to chicks. Altering the microbiota of chicks after hatching is not a new idea. Since the 1970s, research has been conducted into the effectiveness of competitive exclusion cultures (CEC), usually anaerobically cultured bacteria from adult cecal contents, in reducing *Salmonella* infection in chicks (21). With the observation that competitive exclusion was effective only when administered before *Salmonella* challenge (22), the aim became to administer the probiotic as close to hatching as possible. The first report of *in ovo* administration of a probiotic came from Cox et al. (23), who injected an undefined CEC into the air cell of 17 d.i. eggs. This treatment conferred a greater resistance to Salmonella enterica serovar Typhimurium (23). Despite this early success, further results from injecting CEC into eggs have been variable, with reports of reduced hatchability and early mortality with increased disease resistance falling short of antibiotic controls (14, 24–26). As such, it is worth questioning whether injection is the best delivery method for CEC products. Prior to disinfection at hatcheries, which aims to reduce the abundance of pathogenic bacteria which can reduce hatchability and chick performance, the egg has a surface microbiota similar to the composition of the cecal microbiota (27). A topical application of adult cecal bacteria may more accurately replicate the environment in which chickens and their commensals coevolved, where a setting hen would regularly replenish the surface bacteria of the egg. Additionally, a spray application would remove the issue of hatchability caused by injecting probiotics into eggs. A previous experiment explored the ability of a topical application of diluted adult cecal content to affect microbiota development, but little analysis was conducted to determine which amplicon sequence variants (ASVs) were successfully transplanted from the donor material to recipient chicks (28). This is an important question in terms of developing interventions for commercial use. Regulators are unlikely to approve a treatment of unclassified bacteria sourced directly from adult chicken cecal content. Identifying bacterial taxa that are likely to be successfully transplanted by topical application is the first step toward creating an effective topical probiotic which is acceptable to regulators.

## RESULTS

**FIG 1:**
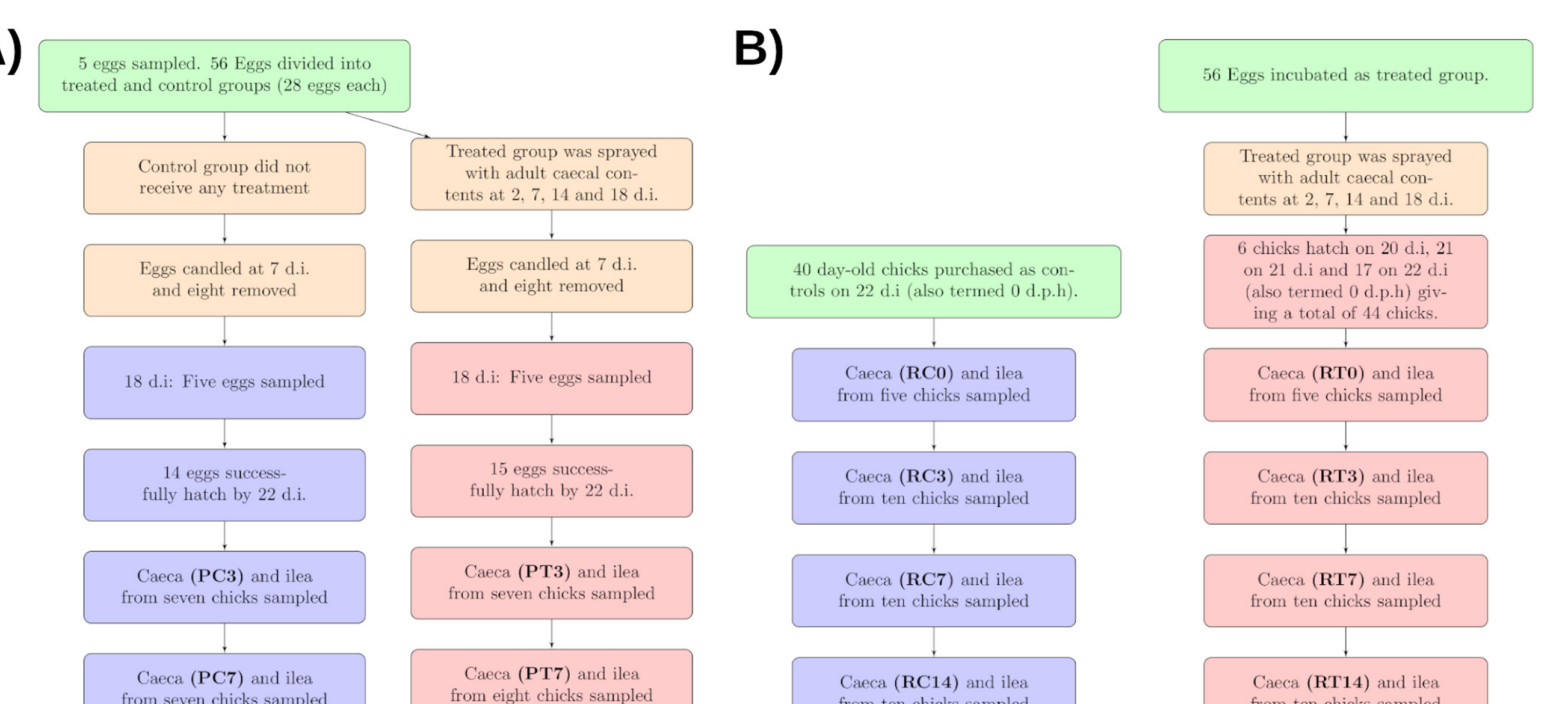
Sampling regimes for the pilot (A) and repeat (B) experiments, including abbreviations for sample groups used when discussing the results.

Two separate experiments, a pilot experiment and a repeat experiment, were conducted to observe the effect of the topical application of adult cecal contents to eggs. Results from both experiments are presented together. A summary of sampling time points and abbreviations can be found in Fig. 1. Briefly, sample groups are identified using abbreviations in which the first letter corresponds to the experiment (P, pilot; R, repeat), the second letter corresponds to the treatment (C, control; T, treated), and the numbers correspond to the time point (0, 3, 7, or 14 days posthatching [dph]). Transplant material is identified by the abbreviation TRPL.

### Sequencing effort.

A total of 22,103,523 reads were obtained from 182 experimental samples submitted for sequencing. After filtering, merging of paired reads, and chimera removal, a total of 15,022,950 reads remained (68% of the original total), giving a mean of 82,544 reads per sample. The median number of reads per sample was 92,218.

### Bacterial 16S rRNA genes were not detected in embryonic samples.

Amplification of standard dilutions revealed that the PCR assay was able to clearly detect 10^3^ bacterial cells in a sample (see Fig. S1 in the supplemental material). No positive amplification of bacterial 16S rRNA genes was detected in any embryonic or egg sample at either 0 or 18 d.i. Amplicons were detected in positive-control samples and all spiked samples, indicating that the absence of amplicons in other samples was not due to PCR failure. This result indicates that no significant population of bacteria was present in the embryonic gut at 18 d.i.

### Treatment had no consistent effect on body weight.

The mean body weights of treated and control chicks in the repeat experiment were compared using Student’s *t* test. No significant differences between groups were found at 0 (treated: mean [M] = 46 g, standard deviation [SD] = 5.83; control: M = 48.4 g, SD = 6.65; conditions: *t* = −0.5, *P* = 0.6), 7 (treated: M = 135 g, SD = 19.0; control: M = 132 g, SD = 17.1; conditions: *t* = 0.35, *P* = 0.7), and 14 (treated: M = 358.5 g, SD = 44.8; control: M = 322.5 g, SD = 39.8; conditions: *t* = 1.8, *P* = 0.13) dph. However, there was a significant difference in body weight between groups at 3 dph (treated: M = 77 g, SD = 7.48; control: M = 65 g, SD = 5.0; conditions: *t* = 4.0, *P* = 0.002).

### Treated chicks had higher alpha diversity at early time points.

**FIG 2:**
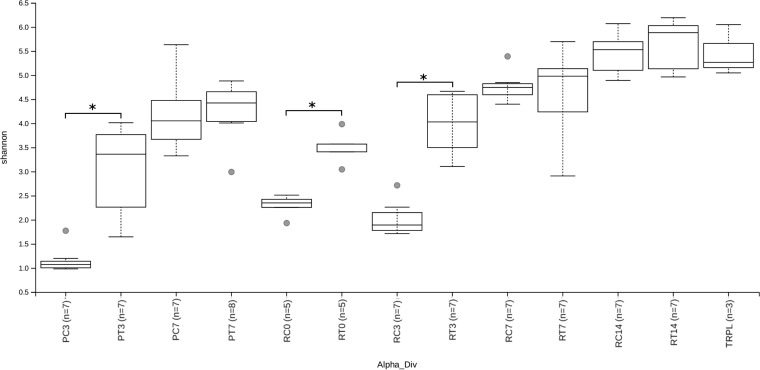
Alpha diversity measured with a Shannon index at a sequencing depth of 5,000. Sample groups divided by experiment (P, pilot; R, repeat), treatment (C, control; T, treated), and age. Shannon diversity of transplant material (TRPL) is also shown. The alpha diversity of treated chicks was significantly higher than that of control chicks at 3 dph (both experiments) and 0 dph (repeat experiment). *, *P* < 0.05.

The alpha diversity of each sample group is displayed in Fig. 2, with the significance of pairwise Kruskal-Wallis tests comparing alpha diversity between sample groups displayed in Fig. S2. Across all experimental groups, alpha diversity increased significantly with age with two exceptions. There was no significant increase in alpha diversity in treated or control chicks between 0 and 3 dph (treated: H = 1.91, *P* = 0.19; control: H = 2.38, *P* = 0.15) or in treated chicks between 3 and 7 dph during the repeat experiment (H = 2.16, *P* = 0.17).

In general, treatment with an adult-derived microbiota resulted in a significantly higher alpha diversity compared to control chicks at 0 dph (repeat: H = 6.82, *P* = 0.017) and 3 dph (pilot: H = 9.02, *P* = 0.009; repeat: H = 9.8, *P* = 0.006) but not 7 and 14 dph. There were significant differences in alpha diversity between transplant material (TRPL) samples and samples taken at 0 and 3 dph as well as PT07 samples.

Control chicks at 3 dph had significantly higher alpha diversity in the repeat experiment than those in the pilot experiment (H = 8.27, *P* = 0.01). There were no further significant differences in alpha diversity between equivalent groups from the pilot and repeat experiments.

### Treatment significantly affected beta diversity.

**FIG 3:**
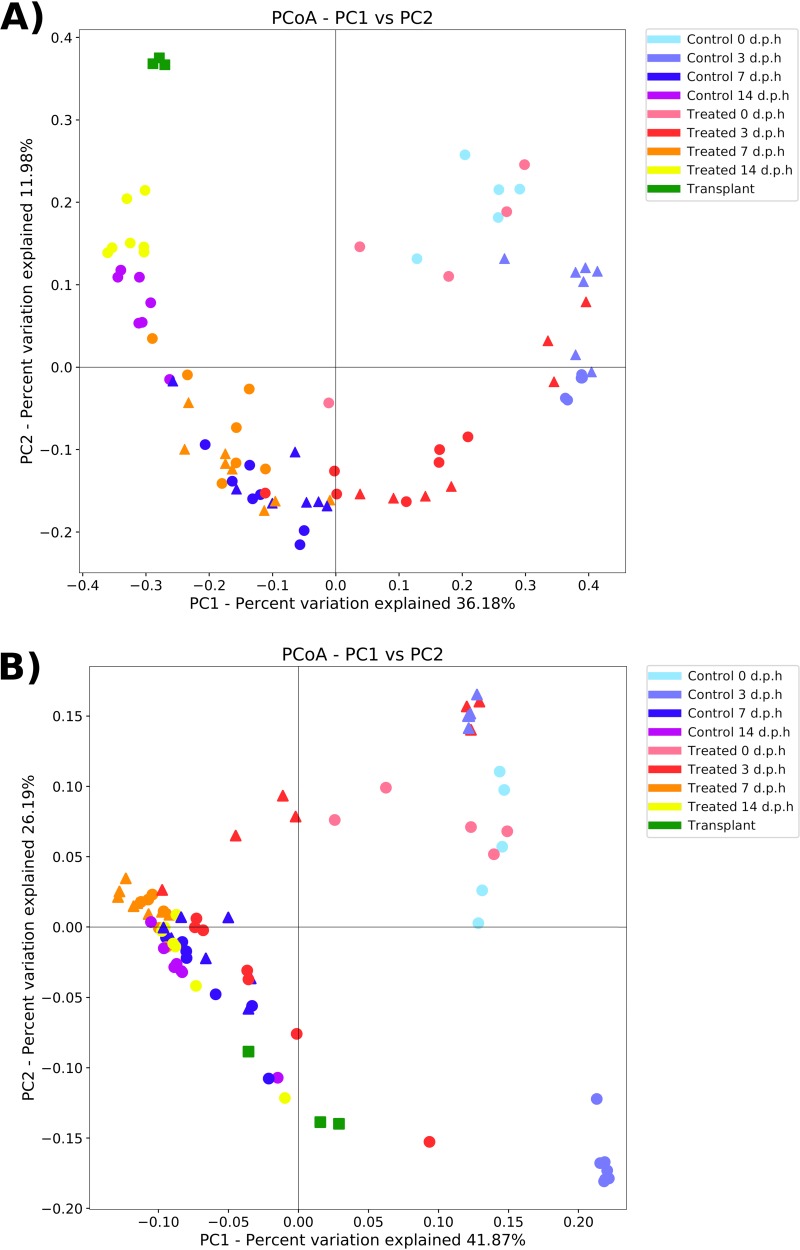
Principal coordinate analysis (PCoA) plot showing differences in unweighted (A) and weighted (B) UniFrac beta diversity between sample groups and treatment groups in pilot (triangle) and repeat experiments (circle) and transplant samples (square). Each point represents an individual sample, with the distance between points representative of differences in microbiota composition.

When measured with an unweighted UniFrac metric, the factor “age” had the largest effect on beta diversity (analysis of similarity [ANOSIM] test statistic = 0.78, *P* = 0.001), followed by “treatment” (ANOSIM test statistic = 0.13, *P* = 0.001) and “experiment” (ANOSIM test statistic = 0.10, *P* = 0.012). The average unweighted UniFrac distance between groups is displayed in Fig. S3. A principal-coordinate analysis (PCoA) analysis showed clustering of samples by group (Fig. 3A). When measured with a weighted UniFrac metric, the factor “age” had the largest effect on beta diversity (ANOSIM test statistic = 0.40, *P* = 0.001), followed by “experiment” (ANOSIM test statistic = 0.16, *P* = 0.002) and “treatment” (ANOSIM test statistic = 0.13, *P* = 0.001). A PCoA analysis showed clustering of samples by group (Fig. 3B).

In plots of unweighted UniFrac distance, RC0 and RT0 tended to cluster together in the PCoA plot, with the exception of one RT0 sample. PC3 and RC3 samples clustered together along with three PT3 samples. The remaining PT3 samples and all RT3 samples clustered together and were closer to samples from later time points than PC3 and RC3 samples. At 7 dph, PC7, PT7, RC7, and RT7 samples clustered together, although there was a tendency for treated samples from both experiments to cluster closer to samples from 14 dph. RC14 and RT14 samples formed separate clusters. A similar pattern of clustering was present in plots of weighted UniFrac distance, although there was no separate clustering of RC14 and RT14 samples. Instead, all samples from 7 and 14 dph tended to cluster together along with RT3 samples and one PT3 sample. Additionally, PC3 and RC3 samples formed distinct clusters compared to the unweighted UniFrac distance plot.

The distance between sample groups and TRPL samples gives some indication of transplant success, as the unweighted UniFrac distance between similar samples is lower, reflecting closer clustering of samples. At 3, 7, and 14 dph, treated samples were significantly closer to TRPL samples than controls (3 dph: *t* = 16.6, *P* < 0.001; 7 dph: *t* = 7.2, *P* < 0.001; 14 dph: *t* = 4.8, *P* < 0.001). This pattern of increased similarity of treated samples to TRPL samples in both experiments suggests that bacteria from adult cecal content successfully colonized chicks by those time points. However, the success of the treatment was not uniform between experiments. PT3 samples are further from TRPL samples than from RT3 samples (cf. 0.76 and 0.66).

### ASVs were differentially abundant between treated and control chicks.

For ease of interpretation, results from the pilot and repeat experiments were interpreted separately. Gneiss analysis was used to identify differentially abundant ASVs between treated and control chicks since it accounts for the compositional nature of microbiome data. First, a dendrogram of ASVs is prepared. Each node is termed a “balance,” with taxa on one side of the balance designated numerators and those on the other, denominators. The log ratio of abundances between numerator and denominator taxa for each balance is calculated. This value can be compared between sample groups to determine differences in microbiome composition. A significant difference between samples indicates that one of the following five hypotheses is true: (i) the numerator taxa are increased in the group with a higher log ratio, (ii) the denominator taxa are decreased, (iii) both hypotheses i and ii are true, (iv) both numerator and denominator taxa are increased, but numerator taxa have increased more, or (v) both numerator and denominator taxa are decreased, but denominator taxa have decreased more. Quantitative PCR is required to discern which hypothesis is correct, as changes in relative abundance are not always reflective of absolute abundance (29).

**FIG 4:**
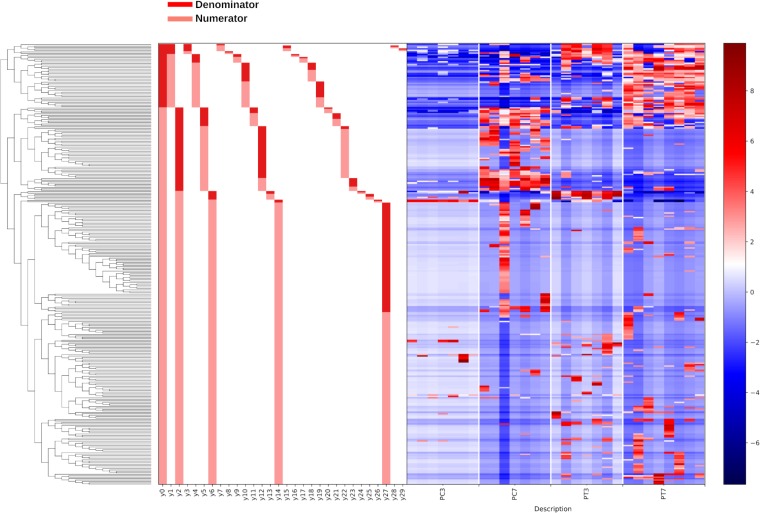
A dendrogram heatmap of ASV log abundance in the cecal microbiota of control and treated chicks at 3 and 7 dph in the pilot experiment. The dendrogram represents the organization of ASVs within the system of balances created by hierarchical clustering. Each node on the dendrogram is a balance, with the first node designated balance y0. Each terminal branch represents an ASV present within the analysis. The bar charts visualize which ASVs are denominator (dark red) and which are numerator (light red) ASVs for each balance. The heatmap shows the log abundance of each ASV in the samples organized by group. Low-abundance ASVs are represented by blue, while higher-abundance ASVs are represented by red.

**(i) Pilot experiment.** Gneiss analysis revealed differential ASV abundance between treated and control chicks at 3 and 7 dph. The ASV table was filtered to exclude ASVs with a total frequency of less than 39 (a justification for filtering thresholds is provided in Materials and Methods), reducing the number of ASVs in the analysis from 408 to 306. The overall linear regression model fit was R2 = 0.34, with the covariate “treatment” accounting for 17.1% of the variance. Log ratio balances y0 (β = −19.8, *P* < 0.001), y2 (β = 9.62, *P* < 0.001), y5 (β = −3.97, *P* = 0.003), y12 (β = −5.42, *P* < 0.001), y14 (β = 6.56, *P* < 0.001), and y27 (β = 7.20, *P* = 0.006) were significant predictors for the covariate “treatment.” On review of the heatmap, balance y6 was considered to describe ASVs differentially present in treated chicks at 3 dph. Fig. 4 shows the log abundance of ASVs at 3 and 7 dph between treated and control chicks along with a summary of balances created by Gneiss analysis. Individual log ratios by group for significant balances and balance taxonomy are available in Fig. S4. The taxonomy of ASVs identified as differentially abundant between treated and control samples is presented in Table 1, with the relative abundance of bacterial families in each sample displayed in Fig. S5. More ASVs assigned to *Lachnospiraceae*, *Bacillaceae*, *Ruminococcaceae*, and *Lactobacillaceae* were found to have a higher relative abundance in treated samples compared to control samples. Some ASVs were found to have a higher abundance in control compared to treated samples and were assigned to *Enterobacteriaceae*, *Erysipelotrichaceae*, and *Peptostreptococcaceae*.

**TABLE 1:** Taxonomy summary at the family level of ASVs identified as higher abundance in treated and control samples from the pilot and repeat experiments using Gneiss analysis

Taxonomy	No. of higher-abundance ASVs			NDA^*a*^
	Total	Treated	Control	
Pilot experiment				
* Lachnospiraceae*	119	80	18	21
* Ruminococcaceae*	104	46	7	51
* Clostridiaceae* 1	21	10	10	1
* Erysipelotrichaceae*	16	4	9	3
* Enterobacteriaceae*	10	3	3	4
* Peptostreptococcaceae*	9	4	5	0
* Bacillaceae*	7	5	0	2
* Clostridiales* vadin BB60 group	6	2	0	4
* Paenibacillaceae*	4	3	1	0
* Enterococcaceae*	3	1	0	2
Uncultured rumen bacterium	2	2	0	0
* Lactobacillaceae*	2	2	0	0
* Christensenellaceae*	1	0	0	1
* Bacillales*	1	1	0	0
* Microbacteriaceae*	1	1	0	0
Repeat experiment				
* Lachnospiraceae*	193	90	20	83
* Ruminococcaceae*	155	79	17	59
* Clostridiaceae* 1	39	1	5	33
* Clostridiales* vadin BB60 group	15	7	1	7
* Erysipelotrichaceae*	12	2	0	10
* Peptostreptococcaceae*	11	3	0	8
* Enterobacteriaceae*	8	0	4	4
* Enterococcaceae*	7	0	3	4
* Bacillaceae*	7	4	0	3
* Lactobacillaceae*	5	2	1	2
* Christensenellaceae*	3	1	0	2
* Paenibacillaceae*	3	0	0	3
* Microbacteriaceae*	2	0	0	2
* Staphylococcaceae*	2	0	0	2
Uncultured rumen bacterium	2	2	0	0
* Thermaceae*	1	0	0	1
* Sanguibacteraceae*	1	0	0	1
* Streptococcaceae*	1	1	0	0
* Hydrogenophilaceae*	1	0	0	1
* Burkholderiaceae*	1	0	0	1
* Propionibacteriaceae*	1	0	0	1
* Leuconostocaceae*	1	0	0	1
* Nocardiaceae*	1	0	0	1
* Peptococcaceae*	1	0	0	1
* Moraxellaceae*	1	0	0	1
* Alicyclobacillaceae*	1	0	0	1

a ASVs identified as NDA were not differentially abundant between treatment groups.

**FIG 5:**
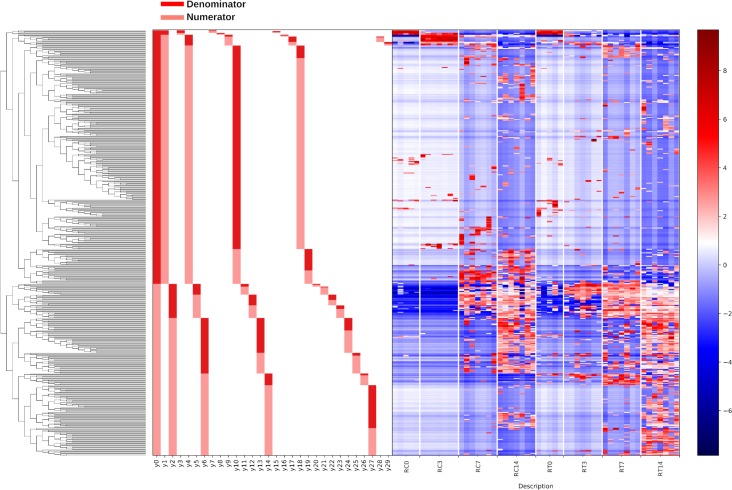
A dendrogram heatmap showing the log abundance of ASVs in the cecal microbiota of control and treated chicks at 0, 3, 7, and 14 dph in the repeat experiment. The dendrogram represents the organization of ASVs within the system of balances created by hierarchical clustering. Each node on the dendrogram is a balance, with the first node designated balance y0. Each terminal branch represents an ASV present within the analysis. The bar charts visualize which ASVs are denominator (dark red) and which are numerator (light red) ASVs for each balance. The heatmap shows the log abundance of each ASV in the samples organized by group. Low-abundance ASVs are represented by blue, while higher-abundance ASVs are represented by red.

**(ii) Repeat experiment.** Gneiss analysis revealed differential ASV abundance between treated and control chicks at 0, 3, 7, and 14 dph. The ASV table was filtered to exclude ASVs with a total frequency of less than 30, reducing the number of ASVs in the analysis from 633 to 475. The overall linear regression model fit was R2 = 0.31, with the covariate “treatment” accounting for 9.65% of the variance. Log ratio balances y0 (β = 14.2, *P* < 0.001), y5 (β = −6.0, *P* = 0.001), y10 (β = −5.7, *P* = 0.009), y14 (β = −7.8, *P* < 0.001), y27 (β = 4.1, *P* = 0.01), and y28 (β = 2.9, *P* < 0.001) were significant predictors for the covariate “treatment.” On review of the heatmap, balance y4 was considered to describe ASVs differentially present in control chicks at 3 dph, and balance y1_denominator_ ASVs were considered to be equally abundant between treated and control samples. Balances y5, y14, y27, and y28 contained ASVs already identified as differentially abundant in treated or control samples by other balances. Figure 5 shows the log abundance of ASVs at 0, 3, 7, and 14 dph between treated and control chicks along with a summary of balances created by Gneiss analysis. Individual log ratios for significant balances and balance taxonomy are available in Fig. S6. The taxonomy of ASVs identified as differentially abundant between treated and control samples is presented in Table 1, with the relative abundance of bacterial families in each sample displayed in Fig. S5. More ASVs assigned to *Lachnospiraceae*, *Ruminococcaceae*, *Clostridiales* vadin BB60 group, *Bacillaceae*, *Peptostreptococcaceae*, and *Mollicutes* RF39 were found to have a higher relative abundance in treated samples compared to control samples. Some ASVs were found to have a higher abundance in control compared to treated samples and were assigned to *Clostridiaceae* 1, *Enterobacteriaceae*, and *Enterococcaceae*.

### Twelve percent of ASVs present in the transplant material were identified as successfully transplanted in the pilot experiment and 20% in the repeat.

A total of 445 ASVs were defined as present in the transplant material. ASVs present in the transplant material that were not subsequently identified in any samples from the pilot and repeat experiments were removed from the analysis (*n* = 274). Most of these ASVs were assigned to *Ruminococcaceae* (*n* = 125), *Clostridiales* vadin BB60 group (*n* = 38), *Lachnospiraceae* (*n* = 33), *Christensenellaceae* (*n* = 12), and *Peptococcaceae* (*n* = 9). ASVs assigned to *Bacteroidaceae*, *Lactobacillaceae*, *Coriobacteriaceae*, *Bifidobacteriaceae*, *Burkholderiaceae*, and *Eggerthellaceae* had a high relative abundance in the transplant material (Fig. S5). However, none of these ASVs were successfully transplanted in either the pilot or repeat experiment.

**(i) Pilot experiment.** A total of 56 ASVs were categorized as successfully transplanted (Table 2; Fig. S7). The taxonomy assignment of ASVs is shown in Table 3. Only ASVs assigned to *Lachnospiraceae*, *Ruminococcaceae*, *Erysipelotrichaceae*, and *Mollicutes* RF39 (uncultured rumen bacteria) were defined as successfully transplanted. A further 49 ASVs were categorized as possibly transplanted, of which the majority were assigned to the families *Ruminococcaceae* and *Lachnospiraceae*. Other ASVs categorized as possibly transplanted were assigned to *Clostridiaceae* 1, *Erysipelotrichaceae*, *Bacillaceae*, *Peptostreptococcaceae*, *Enterobacteriaceae*, *Enterococcaceae*, and *Christensenellaceae*. At the genus level, the two ASVs assigned to *Clostridiaceae* 1 were identified as “Candidatus *Arthromitus*.” The remaining 201 ASVs were categorized as environmental. Some taxa were almost exclusively categorized as environmental, including *Clostridiaceae* 1, *Enterobacteriaceae*, *Peptostreptococcaceae*, *Bacillaceae*, *Clostridiales* vadin BB60 group, *Enterococcaceae*, *Paenibacillaceae*, and *Lactobacillaceae*. A hybrid Sankey diagram showed how the taxonomy of transplanted and environmental ASVs relates to that of ASVs identified as differentially abundant between treated and control samples (Fig. S8A).

**TABLE 2:** Contingency table showing observed frequencies of ASV classification by differential abundance and transplant success in the pilot and repeat experiments^*a*^

ASV classification	Higher abundance in control	Not differentially abundant	Higher abundance in treated	Total
Pilot experiment				
Environmental	40 (31)	63 (58)	98 (112)	201
Possibly transplanted	3 (8)	21 (14)	25 (27)	49
Successfully transplanted	4 (9)	5 (16)	47 (31)	56
Total	47	89	170	
Repeat experiment				
Environmental	41 (37)	206 (171)	102 (141)	349
Possibly transplanted	5 (4)	9 (18)	23 (15)	37
Successfully transplanted	5 (10)	17 (43)	67 (36)	89
Total	51	232	192	

a Expected frequencies calculated using a chi squared test of independence are displayed in parentheses.

**TABLE 3:** Taxonomy at the family level of ASVs defined as successfully transplanted, possibly transplanted and environmental in the pilot and repeat experiments

Taxonomy	No. of ASVs			
	Total	Successfully transplanted	Possibly transplanted	Environmental
Pilot experiment				
* Lachnospiraceae*	119	37	20	62
* Ruminococcaceae*	104	13	21	70
* Clostridiaceae* 1	21	0	2	19
* Erysipelotrichaceae*	16	4	1	11
* Enterobacteriaceae*	10	0	1	9
* Peptostreptococcaceae*	9	0	1	8
* Bacillaceae*	7	0	1	6
* Clostridiales* vadin BB60 group	6	0	0	6
* Paenibacillaceae*	4	0	0	4
* Enterococcaceae*	3	0	1	2
* Lactobacillaceae*	2	0	0	2
Uncultured rumen bacterium	2	2	0	0
* Bacillales*	1	0	0	1
* Christensenellaceae*	1	0	1	0
* Microbacteriaceae*	1	0	0	1
Repeat experiment				
* Lachnospiraceae*	193	45	8	140
* Ruminococcaceae*	155	36	22	97
* Clostridiaceae* 1	39	1	1	37
* Clostridiales* vadin BB60 group	15	1	2	12
* Erysipelotrichaceae*	12	3	0	9
* Peptostreptococcaceae*	11	1	0	10
* Enterobacteriaceae*	8	1	0	7
* Bacillaceae*	7	1	0	6
* Enterococcaceae*	7	0	0	7
* Lactobacillaceae*	5	0	0	5
* Christensenellaceae*	3	0	2	1
* Paenibacillaceae*	3	0	0	3
Uncultured rumen bacterium	2	0	2	0
* Staphylococcaceae*	2	0	0	2
* Microbacteriaceae*	2	0	0	2
* Alicyclobacillaceae*	1	0	0	1
* Propionibacteriaceae*	1	0	0	1
* Hydrogenophilaceae*	1	0	0	1
* Thermaceae*	1	0	0	1
* Peptococcaceae*	1	0	0	1
* Moraxellaceae*	1	0	0	1
* Streptococcaceae*	1	0	0	1
* Leuconostocaceae*	1	0	0	1
* Burkholderiaceae*	1	0	0	1
* Nocardiaceae*	1	0	0	1
* Sanguibacteraceae*	1	0	0	1

**(ii) Repeat experiment.** A total of 89 ASVs were categorized as successfully transplanted (Table 2; Fig. S7B). The taxonomy assignment of ASVs is shown in Table 3. The majority were assigned to the families *Lachnospiraceae* and *Ruminococcaceae*. One ASV that was categorized as successfully transplanted and assigned to *Clostridiaceae* 1 was identified at the genus level as “Candidatus *Arthromitus*.” A further 37 ASVs were categorized as possibly transplanted, the majority of which were assigned to the family *Ruminococcaceae*. The remaining 349 ASVs were categorized as environmental. As for the pilot experiment, some taxa were mainly categorized as environmental, including *Clostridiaceae* 1, *Clostridiales* vadin BB60 group, *Erysipelotrichaceae*, *Peptostreptococcaceae*, *Enterobacteriaceae*, *Bacillaceae*, *Enterococcaceae*, *Paenibacillaceae*, and *Lactobacillaceae*. A hybrid Sankey diagram showed how the taxonomy of transplanted and environmental ASVs relates to that of ASVs identified as differentially abundant between treated and control samples. A hybrid Sankey diagram showed how the taxonomy of transplanted and environmental ASVs relates to that of ASVs identified as differentially abundant between treated and control samples (Fig. S7B).

A contingency table (Table 2) shows the overlap between ASVs identified as differentially abundant and their classification in terms of transplant success in experiments 1 and 2. The relationship between group assignment was significant in the pilot (χ^2^ [4] = 29.2, *P* < 0.001) and repeat (χ^2^ [4] = 72.8, *P* < 0.001) experiments. ASVs identified as differentially abundant in treated chicks were more likely to be defined as successfully transplanted or possibly transplanted.

### Quantitative PCR confirmed differentially abundant taxa between treated and control samples.

**FIG 6:**
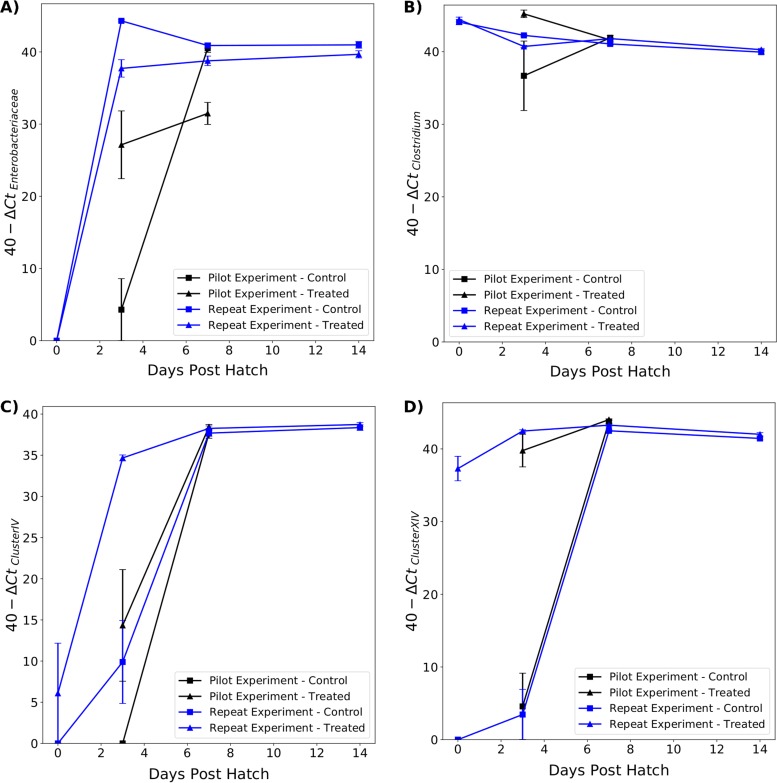
Relative abundance of *Enterobacteriaceae* (A), *Clostridium* (B), *Clostridium* cluster IV (C), and *Clostridium* cluster XIV (a and b) (D) in the ceca of treated and control chicks between 0 and 14 dph. Significant differences between treated and control chicks in the pilot (black lines) and repeat (blue lines) experiments are indicated.

**(i) Cecum.** Genera within the *Lachnospiraceae* have generally been placed in *Clostridium* cluster XIVa, while genera within the *Ruminococcaceae* have generally been placed in *Clostridium* cluster IV (30, 31). As such, primers for *Clostridium* cluster XIV were used to estimate the abundance of *Lachnospiraceae*, and primers for *Clostridium* cluster IV were used to estimate the abundance of *Ruminococcaceae*. The results are presented in Fig. 6.

The abundances of *Clostridium* cluster XIV were significantly different in RT0 samples (*t* = 22.14, *P* < 0.001), as no DNA was amplified using this primer in RC0 samples. In both experiments, treated chicks had significantly more *Clostridium* cluster XIV at 3 dph (pilot: *t* = 7.24, *P* < 0.001; repeat: *t* = 11.3, *P* < 0.001). The difference between treated and control chicks continued to be significant in the repeat experiment at 7 dph (*t* = 4.0, *P* < 0.001) but not in the pilot experiment. There was no significant difference in *Clostridium* cluster XIV abundance between the groups at 14 dph.

There was no significant difference in *Clostridium* cluster IV abundance between RC0 and RT0 samples, as DNA from these taxa was amplified in only one RT0 sample. The abundance of *Clostridium* cluster IV was significantly higher in RT3 samples than RC3 samples (*t* = 4.9, *P* < 0.001), but the result was not quite significant when comparing PT3 and PC3 samples (*t* = 1.95, *P* = 0.07). By 7 and 14 dph, there was no significant difference in *Clostridium* cluster IV abundance between treated and control chicks.

There were differences in abundance of *Clostridium* cluster IV between treated chicks from the pilot and repeat experiments. RT3 samples had a higher abundance of *Clostridium* cluster IV than PT3 samples (*t* = 3.63, *P* = 0.002).

No *Enterobacteriaceae* were detected in either RT0 or RC0 samples. There was a significantly lower abundance of *Enterobacteriaceae* in RT3 samples compared to RC3 samples (*t* = −5.42, *P* < 0.001). However, in the pilot experiment, the opposite result was obtained, with a significantly higher abundance of *Enterobacteriaceae* in PT3 samples than PC3 samples (*t* = 3.54, *P* = 0.005). At 7 dph, there was a significantly lower abundance of *Enterobacteriaceae* in treated chicks in both experiments (pilot: *t* = −5.24, *P* < 0.001; repeat: *t* = −2.85, *P* = 0.01). On average, the abundance of *Enterobacteriaceae* was lower in RT14 samples than in RC14 samples, but the difference was not significant (*t* = −1.95, *P* = 0.07). There was a large interexperiment variation in *Enterobacteriaceae* abundance, with higher abundance detected at 3 dph in the repeat experiment.

High levels of *Clostridium* were detected in RT0 and RC0 samples, and there were no significant differences between the groups. The abundance of *Clostridium* was significantly lower in RT3 samples than in RC3 samples (*t* = −7.78, *P* < 0.001) but was significantly higher in RT7 samples than in RC7 samples (*t* = 3.5, *P* = 0.002). There were no significant differences in *Clostridium* abundance between treated and control chicks in the pilot experiment or in the repeat experiment at 14 dph.

**FIG 7:**
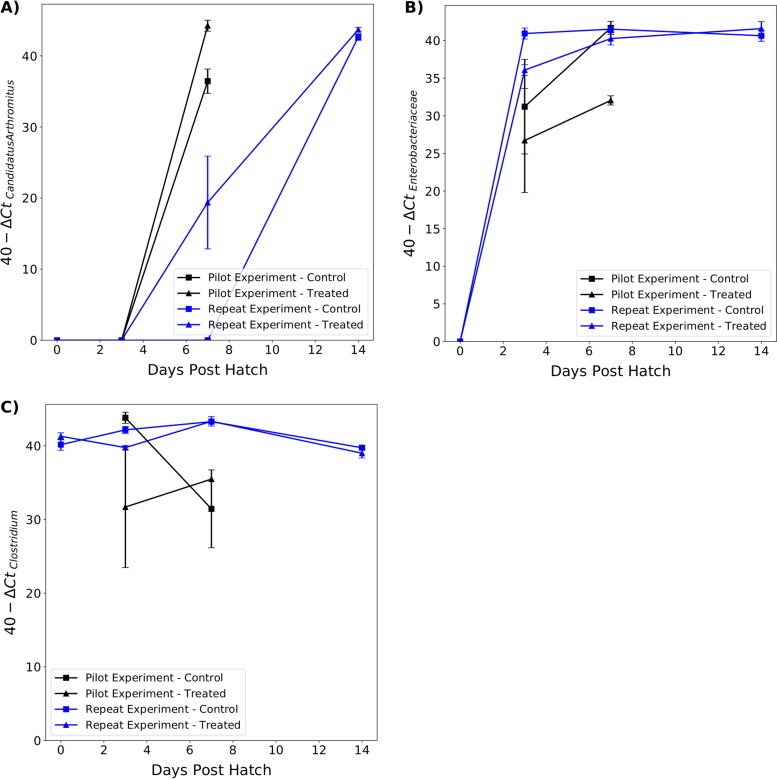
Relative abundance of “Candidatus *Arthromitus*” (A), *Enterobacteriaceae* (B), and *Clostridium* (C) in the ilea of treated and control chicks between 0 and 14 dph. Significant differences between treated and control chicks in the pilot (black lines) and repeat (blue lines) experiments are indicated.

**(ii) Ileum.** No *Enterobacteriaceae* were detected in treated or control chicks in the repeat experiment at 0 dph (Fig. 7). There was a significantly lower abundance of *Enterobacteriaceae* in treated chicks than in control chicks at 3 dph in the repeat experiment (*t* = −4.78, *P* < 0.001) and 7 dph in the pilot experiment (*t* = −9.27, *P* < 0.001). There were no significant differences between treated and control chicks at other time points.

There was no significant difference in *Clostridium* abundance between treated and control chicks at any time point except at 3 dph in the repeat experiment, where the abundance was lower in treated chicks (*t* = −4.33, *P* < 0.001).

In both experiments, no “Candidatus *Arthromitus*” was present in the ileum until 7 dph. At 7 dph, the abundance of “Candidatus *Arthromitus*” was significantly higher in treated chicks in the pilot and repeat experiments (*t* = 4.35, *P* < 0.001 and *t* = 2.97, *P* = 0.008, respectively). Although the average abundance of “Candidatus *Arthromitus*” was higher in treated chicks from the repeat experiment at 14 dph, the difference was not significant (*t* = 1.88, *P* = 0.08).

### Treatment did not alter intestinal morphology.

Histological examination of ileal and cecal tonsil tissues from the repeat experiment at 0, 3, 7, and 14 dph was conducted to observe morphological parameters associated with intestinal development such as villus height and width, epithelial cell height, and crypt mitotic figure counts.

Significantly more mitotic figures (Fig. S9A and B) were recorded in the crypts of control chicks than treated chicks at 3 dph in the ileum (*t* = 3.53, *P* = 0.008) and cecal tonsil (*t* = 2.81, *P* = 0.03). There was no statistically significant difference in mitotic figure counts (F) between age groups in the ileum (F = 3.1, *P* = 0.09) and cecal tonsil (F = 0.18, *P* = 0.94).

There were no significant differences in epithelial cell height, villus height, or villus width between treated and control chicks at 0, 3, 7, or 14 dph (Fig. S9C to E). There was a statistically significant difference in villus height (F = 41.6, *P* < 0.001), villus width (F = 18.1, *P* < 0.001), and epithelial cell height (F = 58.9, *P* < 0.001) between age groups. *Post hoc* comparisons revealed that villus height was significantly different between 0 and 3 dph (*P* = 0.001), 3 and 7 dph (*P* = 0.001), and 7 and 14 dph (*P* = 0.001). Villus width was significantly different between 0 and 3 dph (*P* = 0.01) and 7 and 14 dph (*P* = 0.001) but not between 3 and 7 dph (*P* = 0.75). Epithelial cell height was significantly different between 0 and 3 dph (*P* = 0.001) and 3 and 7 dph (*P* = 0.001) but not between 7 and 14 dph (*P* = 0.66).

Segmented filamentous bacteria were observed in the ileum from 7 dph, with the presence on ileal histology correlating with the presence of “Candidatus *Arthromitus*” detected by quantitative PCR (qPCR). In the repeat experiment, segmented filamentous bacteria were also seen in the cecal tonsil in close approximation to epithelial cells and in the lumen (Fig. S10A).

No bacteria were found in the cecal crypts of any chicks at 0 dph or control chicks at 3 dph; however, bacteria were identified in the cecal crypts of four treated chicks (Fig. S10B). At 7 dph, four treated chicks and six control chicks were positive. At 14 dph, no treated or control chicks were positive, although occasional bacteria were noted in cecal crypts.

### Treatment did not affect immune cell populations in the cecal tonsil.

Tissue from chicks at 3 dph was examined, as this was the time point when most differences were found between the microbiota of treated and control chicks. No significant differences in counts of CD4, CD8α, CD8β, γδ T-cell receptor (TCR), or Bu1 cells were found between treated and control cecal tonsils at 3 dph.

## DISCUSSION

This study demonstrated that there were no detectable bacteria present in the embryonic gut at 18 d.i. It could be argued that detection of PCR amplicons using gel electrophoresis was not sensitive enough to detect small numbers of bacteria within the embryonic gut. It could be expected that a bacterial population too small to be detected using PCR would also be too small to have an impact in the face of overwhelming colonization by other bacterial taxa at hatching. It is likely that the chicken gut remains sterile until hatching, when bacteria present on the egg surface are the first to colonize.

Inoculation of the egg surface with an adult-derived microbiota was sufficient to transfer elements of the microbiota to chicks, with the result of accelerating cecal microbiota development. In treatment and control groups across both experiments, microbial succession followed the same pattern. The microbiota of day-old chicks was poorly diverse and composed of environmental bacteria, a pattern well described in current literature (13, 28, 32). The order of succession whereby environmental bacteria were replaced first by *Lachnospiraceae* and then by *Ruminococcaceae* and other *Clostridiales* was common across treated and control chicks. However, the speed of succession was faster in treated chicks in both experiments, with an initial strong colonization by *Lachnospiraceae* followed by an increase in *Ruminococcaceae*. Many *Ruminococcaceae* ASVs were classified as successfully or possibly transplanted, suggesting that these ASVs were present at 0 and 3 dph but were unable to colonize the cecum initially. This suggests that alterations to cecal conditions by *Lachnospiraceae* or some other unknown host factor are a prerequisite for colonization by *Ruminococcaceae*. These results are in contrast to those of Donaldson et al. (28), who did not observe significant differences in alpha diversity or the pattern of bacterial colonization between treated and control birds. These differing results can be explained by different techniques used to apply the transplant material. Donaldson et al. (28) swabbed the egg surface with diluted adult cecal content once during incubation, which may have resulted in the application of lower numbers of spores and vegetative cells to the eggshell than a spray. Additionally, multiple treatments may have allowed for an accumulation of viable bacterial spores more akin to the effect of close contact with the hen during incubation.

The presence of ASVs common to the transplant material from as early as a few hours posthatching shows that the cecal microbiota can be successfully transplanted to chicks by topical application to the egg surface. Most of the successfully transplanted ASVs were assigned to *Lachnospiraceae* and *Ruminococcaceae*; this differs from previously published results. Pedroso et al. (14) found that only one operational taxonomic unit assigned to *Lachnospiraceae* was transferred to treated chicks after inoculating eggs with an *in ovo* injection of a commercial probiotic competitive exclusion (CPCE) product. In contrast, we found that the majority of transferred features were assigned to *Lachnospiraceae*, *Ruminococcaceae*, and other *Clostridiales*. CPCE products are collections of culturable bacteria, but the results of our study show that the ASVs most likely to successfully colonize and persist within the chicken cecum belonged to taxa that are challenging to culture in the laboratory, such as *Lachnospiraceae*, *Ruminococcaceae*, and to a lesser degree, *Clostridiales* vadin BB60 and *Mollicutes* RF39. It is unlikely that current CPCE products have an optimal bacterial composition for long-term colonization of chicks. Development of CPCE products should focus on including the aforementioned bacterial taxa, as these have been shown to readily colonize newly hatched chicks and persist within the cecum. However, topical application of cecal contents was unable to transplant several important taxa, such as *Bacteroidaceae*, *Lactobacillaceae*, and *Bifidobacteriaceae*. A recent study of whole-genome sequences from cecal bacteria revealed that genes enabling sporulation were found within most Gram-positive *Firmicutes*, such as *Lachnospiraceae* and *Ruminococcaceae*, with the exception of *Lactobacillaceae* (33). No *Bacteroidetes* isolates were spore-forming; however, 73% of *Bacteroidetes* isolates were microaerotolerant and able to survive air exposures of 24 h (33). Similarly, the bifidobacteria isolated from the chicken gut are non-spore-forming (34). This difference in environmental survival strategy explains the pattern of transplanted features observed in this study. Spores would be able to survive on the egg surface and colonize the chick at hatching, whereas non-spore-forming members of the cecal microbiota would not survive the 72 h from the last treatment to hatching. Alternatively, since bacterial viability was not assessed in the transplant material, the storage and handling of the cecal content may have negatively impacted the survival of taxa that were not transplanted. The inability of a topical treatment of diluted adult cecal content at 18 days of incubation to transfer *Bacteroidaceae*, *Lactobacillaceae*, and *Bifidobacteriaceae* exposes a major weakness of the technique, whether that is due to oxygen exposure during treatment or reduced viability due to storage. *Bacteroidaceae* are considered core members of the chicken cecal microbiota (12, 35, 36), while members of the *Bifidobacteriaceae* have been positively correlated with increased bird weight (35). Any future experiments aiming to transplant an adult cecal microbiota would need to take these taxa into account by delivering treatments immediately after hatching, either directly to the chick or into the environment. Exploring different methods of bacterial preservation by using more appropriate storage media to improve bacterial viability provides another avenue for future investigation.

The abundance of “Candidatus *Arthromitus*,” also known as “Candidatus *Savagella*” or segmented filamentous bacteria was studied in the ileum due to its importance as an immunostimulatory bacterium (37–39). Consistent with previous experiments, “Candidatus *Arthromitus*” was absent from the ileum until 7 dph (40), after which a higher abundance was present in treated chicks. The environmental factors influencing “Candidatus *Arthromitus*” colonization have not been explored, although increased abundance was noted in the ilea of chicks housed on reused litter (41). The transplant material may have contained “Candidatus *Arthromitus*” spores that transferred to treated chicks, resulting in a higher abundance once ileal conditions were suitable for colonization. Alternatively, the presence of transplanted bacteria in treated chicks may have created favorable metabolic or immunological conditions allowing earlier and greater colonization by “Candidatus *Arthromitus*.” Treatments which result in early colonization by “Candidatus *Arthromitus*” should be of interest to poultry producers, as earlier colonization by “Candidatus *Arthromitus*” has been positively correlated with body weight (35). Segmented filamentous bacteria were found in the cecal tonsil on histology. The presence of segmented filamentous bacteria in close approximation to the cecal tonsil epithelium was previously reported in 1978 (42). While previous studies have been conducted to investigate the role of segmented filamentous bacteria on immune development in the ileum of mice (38, 43), no studies have focused on similar effects in either the ileum or the cecal tonsil of chickens.

The transplant was more successful in the repeat experiment as evidenced by improved early transplant uptake and persistence of significant differences in alpha diversity until 7 dph. The reason for this variability is hard to assess. Since the storage and application of transplant material were uniform across the two experiments, uncontrolled variables such as the initial microbiota or other environmental bacteria may have affected transplant success.

A potential use for cecal microbiota transplants in chicks is the competitive exclusion of potential pathogens such as *Enterobacteriaceae* and *Clostridium* during the first week posthatching. In the repeat experiment, there was a significant difference in the colonization of *Enterobacteriaceae*, with a consistently lower abundance in treated chicks. The role of some *Enterobacteriaceae* in the chicken cecal microbiota is unclear. While Escherichia coli has the potential for pathogenicity, it is often found in the ceca of healthy chickens. As such, the higher abundance of *Enterobacteriaceae* in control chicks may not be a cause for concern. However, large blooms of *Enterobacteriaceae* unopposed by other taxa, such as that in control chicks from the repeat experiment, are unlikely to be beneficial to the host. In this regard, the transplant was successful, as a similar overgrowth of *Enterobacteriaceae* was avoided in treated chicks. The lower abundance of *Enterobacteriaceae* in treated chicks is likely due to the presence of short-chain fatty acid (SCFA)-producing bacteria such as *Lachnospiraceae* and *Ruminococcaceae*. Previous studies have found an inhibitory effect of SCFAs on *Enterobacteriaceae* growth both *in vitro* and *in vivo* (44). The treatment had less of an impact on the abundance of *Clostridium*. The most significant species of *Clostridium* in terms of chicken health is Clostridium perfringens, which has been linked to necrotic enteritis in chicks. Direct challenges using this species and other more significant pathogens, such as *Campylobacter* and *Salmonella*, are required to further explore how cecal microbiota transplants can affect pathogen abundance in the cecum.

This study did not find statistically significant differences in intestinal morphology between treated and control chicks, except that the mitotic figure count was higher in both the ileum and the cecum of control chicks at 3 dph. The cecal microbiota of treated and control chicks at this time point were markedly different, with control chicks mainly colonized by *Enterobacteriaceae*. If mitotic figure count is reflective of epithelial cell replacement rates, this could imply that the presence of *Enterobacteriaceae* increased epithelial cell replacement. Equally, it could be argued that the lack of *Lachnospiraceae* and *Ruminococcaceae* may have induced higher epithelial cell turnover in control chicks since the bacterial metabolite butyrate decreases apoptosis of normal enterocytes (45). Body weights were also significantly different between treated and control chicks at 3 dph. As with ileal epithelial turnover, if this difference was attributable to the microbiota, it is not possible to distinguish if the cause was a negative effect of *Enterobacteriaceae* or a positive effect of *Lachnospiraceae* and *Ruminococcaceae*.

No significant differences in immune cell populations were found between treated and control chicks at 3 dph despite large differences in the cecal microbiota. While these differences did not have an impact on the number of immune cells in the cecal tonsil, it remains possible that the presence of different bacterial species stimulates differential gene expression in immune cells since a role of SCFA-producing bacteria in immune development has been studied in other species (46, 47).

The presence of bacteria in cecal tonsil crypts has not previously been reported in chickens. This observation was age dependent, with sparse bacteria observed at 14 dph compared to 3 and 7 dph. This raises the prospect that the presence of bacteria in the cecal tonsil crypts has some role in immune development which subsequently excludes them from this niche. It was not possible to determine the taxonomy of these bacteria; however, it is likely that they were *Lachnospiraceae* or *Ruminococcaceae* due to the absence of bacteria in the cecal tonsil crypts of all chicks at 0 dph and control chicks at 3 dph. Additionally, a previous study found that these taxa have a higher relative abundance in cecal mucus compared to lumen contents (48).

In summary, three topical applications of dilute adult cecal content to the eggshell were sufficient to transplant elements of the cecal microbiota to newly hatched chicks, resulting in accelerated development of the cecal microbiota. However, while important members of the cecal microbiota, such as *Lachnospiraceae* and *Ruminococcaceae*, were successfully transplanted, topical application failed to transplant *Bacteroidaceae* or *Lactobacillaceae*. Topical application of characterized bacterial communities to the eggshell during incubation provides a mechanism to transfer a desirable intestinal microbiota to chicks and reduce colonization by possible pathogens. However, treatment ending at 18 d.i. successfully transferred only spore-forming bacteria, and further experiments are required to determine whether non-spore-forming microbiota can be transplanted by topical treatments in the hours before or after hatching.

## MATERIALS AND METHODS

### Animals and housing.

**(i) Pilot experiment.** A total of 61 Ross 308 eggs were purchased from a local hatchery (Annyalla Chicks, Wrexham, UK). Hatchery eggs were disinfected daily during storage using a fog application of Virocid (Cid Lines), a disinfectant based on quaternary ammonium, glutaraldehyde, and isopropanol. Eggs at the hatchery were disinfected further using formaldehyde fumigation before being set. On arrival at the experimental housing, 5 eggs were selected for sampling at 0 d.i. The remaining 56 eggs were divided into a treatment group and a control group of 28 eggs each. Each group was housed in different incubators in different rooms. A biosecurity protocol was implemented whereby the control group was handled first to avoid transfer of environmental bacteria from the treatment to the control group. Eggs were incubated at 37.5°C for 21 days. The eggs were candled at 7 d.i. to assess viability. In both groups, 8 eggs were removed, as no embryonic development had occurred. From each group, 5 eggs were removed for sampling at 18 d.i. The remaining 15 eggs in each group were left to hatch. In total, 15 and 14 chicks hatched from the treatment and control groups, respectively. Chicks were left in the incubators until dry, before being transferred to brooder pens with a wood shaving substrate. Water and feed were provided *ad libitum* by a drinker and feeder present in each brooder. Chicks were fed a vegetable protein-based starter diet for the duration of the experiment (Table 4). Seven chicks from each group were sampled at 3 days posthatching (dph), with the remaining 8 treated and 7 control chicks sampled at 7 dph. No unexpected deaths occurred in either group over the course of the experiment.

**TABLE 4:** Composition of starter and grower diets

Analytical constituents	Diet	
	Starter (mg/kg)	Grower (mg/kg)
Crude fat	2.7	2.4
Crude protein	18.9	15.6
Crude fiber	3.8	4.1
Crude ash	6.6	5.6
Lysine	0.99	0.69
Methionine	0.44	0.27
Calcium	1.05	0.89
Phosphorus	0.7	0.62
Sodium	0.15	0.15
Magnesium	0.17	0.22
Copper	15	16

**(ii) Repeat experiment.** A total of 56 Ross 308 eggs were purchased from a local hatchery (Annyalla Chicks, Wrexham, UK). Eggs underwent the same disinfection procedure at the hatchery as described for the pilot experiment. On arrival at the experimental housing, the 56 eggs were divided between two incubators of 28 eggs each. The day that incubation started was defined as 0 d.i. Both incubators were housed in the same room. Eggs were incubated at 37.5°C for 22 days. The eggs were candled at 7 d.i. to assess viability. Eggs began to hatch at 20 d.i., with 6 chicks hatching at 20 d.i., 21 at 21 d.i. and 17 at 22 d.i., giving a total of 44 chicks. After hatching, chicks were left in the incubators until dry before being transferred to brooder pens with a wood shaving substrate. At 22 d.i., 40 day-old chicks were purchased from the same hatchery. These chicks were the control group and were housed separately from treated chicks. Water and feed were provided *ad libitum* by a drinker and feeder present in each brooder. Chicks were fed a vegetable protein-based starter diet for the duration of the experiment. Five chicks from each group were sampled on the same day the control chicks were brought to the housing (defined as 0 dph). Ten chicks from each group were sampled at 3, 7, and 14 dph. Two chicks from the treatment group died unexpectedly during the experiment, one at 1 dph and another at 6 dph. The cause of death was not determined, although a preliminary gross postmortem examination revealed peritonitis and perihepatitis, consistent with early opportunistic bacterial infection.

**(iii) Treatment.** Entire cecal contents were collected from healthy 42-day-old chickens from three different breeds (Ross 308, Hubbard JA87, and Cobb 500) as part of an experiment to observe the normal development of the cecal microbiota (48). A total of 200 mg of cecal contents from five individuals of each breed was pooled, and DNA was extracted for sequencing. The remaining cecal contents were stored at –20°C for 14 months. Before experimental work began, cecal contents from Ross, Cobb, and Hubbard birds were defrosted, mixed, and diluted 1:20 in sterile phosphate-buffered saline. Aliquots of 5 ml of diluted cecal content were prepared and frozen at –20°C for use as treatments. Treatment group eggs were sprayed at 2, 7, 14, and 18 d.i. in both trials. The diluted cecal contents were defrosted at room temperature and loaded into a 10-ml spray bottle. Eggs were sprayed evenly at a distance of 10 cm, ensuring that all eggs received at least two sprays until the 5 ml of diluted cecal content had been used.

### Sample collection.

**(i) Pilot experiment.** Samples were taken from eggs at 0 d.i. To minimize the risk of contamination, eggs were sprayed with 70% ethanol and left for 10 minutes before being wiped clean. Samples were taken in as sterile an environment as possible. All samples were taken inside an exclusion cabinet, and sterile gloves were worn and changed between eggs. An electric rotary tool (Dremel 3000) was used to cut through the eggshell without penetrating the shell membranes. A sterile scalpel was used to cut the shell membrane to remove the top of the eggshell and reveal the yolk. Sterile needles and syringes were used to sample from the albumen and the yolk.

At 18 d.i., samples were taken from five embryos from each group. Embryos were killed by refrigerating the egg at 3°C for 4 hours. The eggshell was opened as previously described. A sample of amniotic fluid was taken using a sterile needle and syringe. The embryo was removed from the egg, placed in a sterile petri dish, and placed under a stereomicroscope for dissection. The coelom was opened with a sterile scalpel and forceps to reveal the gastrointestinal tract, which was removed. The duodenum, jejunum, and ileum were stored together, with the two ceca stored in separate containers. Finally, the brain was removed using a new sterile scalpel to be used as a control for contamination should bacterial DNA be recovered from the gastrointestinal tract.

Further samples were taken at 3 and 7 dph. Chicks were euthanized by cervical dislocation. To sample chicks, the abdomen was sprayed with 70% ethanol. Skin incisions were made to expose the sternum, which was then reflected to give good access to the coelom. The gastrointestinal tract was removed carefully to avoid external contamination. The ileum, defined as the intestinal segment from Meckel’s diverticulum to the ileocecocolic junction, and the two ceca were removed and stored in separate containers. Samples for DNA extraction were snap-frozen in liquid nitrogen and stored at −20°C.

**(ii) Repeat experiment.** The same sampling protocol was used as for the pilot experiment, with chicks sampled at 0, 3, 7, and 14 dph. After euthanasia, chicks were weighed and their body weights recorded in grams. Additionally, tissue samples from the cecal tonsils, identified as the proximal section of the cecum, and the ileum were taken. One cecal tonsil and a section of ileum were fixed in 4% paraformaldehyde solution for histological examination. The other cecal tonsil was fixed in OCT embedding matrix (CellPath, UK) on a cork plate and snap-frozen in liquid nitrogen. Samples fixed in paraformaldehyde were stored at 4°C, samples for DNA extraction were stored at –20°C, and samples fixed in OCT were stored at –80°C.

### DNA extraction.

DNA was extracted from each sample using ZymoBIOMICS DNA minikits (Cambridge Bioscience, UK) according to the manufacturer’s instructions. DNA was extracted from 250 μl of liquid samples (albumen and yolk). For tissue samples (ileum and cecum), a 200-mg section of intestinal tissue along with content was used for DNA extraction. This section was cut longitudinally and transversely using a sterile scalpel blade to expose the mucosa and luminal contents to bead-beating. Both liquid and tissue samples underwent a bead-beating step with a Qiagen TissueLyser at 30 Hz for 10 minutes. At each extraction, two controls were included, a blank extraction kit to control for contamination and 75 μl of ZymoBIOMICS standard bacterial community (Cambridge Bioscience, UK) to control for variations in DNA extraction efficacy. Extracted DNA was quantified using a NanoDrop 2000 spectrophotometer (NanoDrop Technologies).

### PCR to detect bacterial DNA.

The detection of bacterial DNA in egg and embryonic samples was performed using PCR detection of the bacterial 16S rRNA gene. Purified DNA from egg and embryonic samples from the pilot experiment was used as the template in a PCR mixture composed of 5 μl of 5× FIREPol master mix ready to load (Solis BioDyne, Estonia), 1 μl of each primer, 17 μl of purified water, and 1 μl of DNA template. A primer pair spanning the V4 region of the 16S rRNA gene (515F, TGCCAGCMGCCGCGGTAA; R806, GGACTACHVGGGTWTCTAAT) was used (49). DNA extracted from the ZymoBIOMICS standard bacterial community (ZSBC), which contains approximately 1.4 × 10^10^ cells/ml, was used as a positive control. Thermal cycling consisted of an initial cycle of 95°C for 5 min, 30 cycles of 95°C for 30 s, 55°C for 45 s, and 72°C for 40 s followed by a final cycle of 72°C for 40 s. The presence of PCR products was confirmed with electrophoresis using a 1.0% agarose gel containing ethidium bromide. To exclude the possibility that negative results were due to PCR inhibitors present within samples, 9 μl of each sample was spiked with 1 μl of DNA extracted from the ZSBC and submitted for PCR amplification. To determine the sensitivity of the PCR assay, DNA extracted from the ZSBC was diluted to include the equivalent of DNA extracted from 10^6^, 10^5^, 10^4^, 10^3^, 10^2^, and 10^1^ bacterial cells.

### Illumina MiSeq sequencing.

Extracted DNA from five to eight cecal samples in each treatment group at each time point was sent for paired-end sequencing of the 16S rRNA gene at the Centre for Genomic Research (University of Liverpool) using an Illumina MiSeq run. The V4 hypervariable region (515F/R806) was amplified for 25 cycles to yield an amplicon of 254 bp (50). Library preparation was performed using a universal tailed tag design, with subsequent amplification performed using a two-step PCR with a HiFi Hot Start polymerase (Kapa) (51). The first round of PCR was performed using the primers 5′-ACACTCTTTCCCTACACGACGCTCTTCCGATCTNNNNNGTGCCAGCMGCCGCGGTAA-3′ (forward) and 5′ GTGACTGGAGTTCAGACGTGTGCTCTTCCGATCTGGACTACHVGGGTWTCTAAT-3′ (47). The raw FASTQ files were trimmed for the presence of Illumina adapter sequences using Cutadapt version 1.2.1. The reads were further trimmed using Sickle version 1.200 with a minimum window quality score of 20. Reads shorter than 10 bp after trimming were removed. Raw sequence reads are available in the NCBI Sequence Read Archive under BioProject PRJNA517619.

### Data analysis.

QIIME2 version 2019.1.0 was used for analysis of the Illumina data (52). Amplicon sequence variant (ASV) assignment was completed using the dada2 plug-in (53) and an ASV table produced in biological observation matrix (BIOM) format (54). The resulting ASV table was divided into three tables, one containing all samples, including transplant samples for use in diversity analyses, and one each for samples from the pilot and repeat experiments to identify differentially abundant ASVs between control and treatment groups. Taxonomy was assigned using the q2-feature-classifier plug-in (55) with a pretrained NaiveBayes classifier based on the SILVA 132 database of the 515F/R806 region of the 16S rRNA gene (56), available for download at https://docs.qiime2.org/2018.11/data-resources/.

Alpha and beta diversity analyses were performed at a sampling depth of 5,000 using the alignment (57), phylogeny (58), and diversity (https://github.com/qiime2/q2-diversity) plug-ins. Alpha diversity, a metric used to assess species richness and evenness, was measured using a Shannon diversity index. Taxa plots were produced using the q2-taxa plug-in (https://github.com/qiime2/q2-taxa). Beta diversity, a metric used to compare species diversity and abundance between samples, was calculated with unweighted and weighted UniFrac metrics.

### Statistics.

For statistical analysis, samples were grouped according to age, treatment group, and experiment, creating the following 13 groups for comparison: samples from the pilot experiment from control (C) and treated (T) chicks at 3 and 7 dph (PC3, *n* = 7; PT3, *n* = 7; PC7, *n* = 7; PT7, *n* = 8), samples from the repeat experiment from control (C) and treated (T) chicks at 0, 3, 7, and 14 dph (RC0, *n* = 5; RT0, *n* = 5; RC3, *n* = 7; RT3, *n* = 7; RC7, *n* = 7; RT7, *n* = 7; RC14, *n* = 7; RT14, *n* = 7), and transplant material (TRPL, *n* = 3). Alpha diversities were compared between groups using a pairwise Kruskal-Wallis test with a false discovery rate correction. An ANOSIM test was used to identify metadata categories which significantly affected beta diversity. The average distances from samples in each group to TRPL samples were compared using an independent Student *t* test to find which group was closest to TRPL samples. Gneiss analysis (29) was used to identify taxa which were differentially abundant between treatment and control groups in the pilot and repeat experiments separately. First, the ASV table was filtered to exclude transplant samples and low abundance ASVs. The count threshold for exclusion of ASVs was set at the first quartile to exclude the lowest 25% of ASVs by total frequency across all samples. Principal balances for use in the Gneiss analysis were obtained via Ward’s hierarchical clustering using the correlation-clustering command. Log ratios for each balance were calculated using the ilr-transform command. A multivariate response linear regression model of log ratio balances was constructed with treatment and days posthatching as covariates using the ols-regression command. Results were visualized through a regression summary, dendrogram heatmaps and balance taxonomies to identify ASVs which were differentially abundant in treated and control groups. Based on this analysis, ASVs were divided into three groups, ASVs with a higher relative abundance in treated samples, ASVs with a higher relative abundance in control samples, and ASVs with no differential abundance between groups. The results of this analysis were used to select taxa for further analysis using quantitative PCR.

### Identifying ASVs transplanted from the treatment.

ASVs present in an unfiltered ASV table of TRPL samples were defined as being present in the transplant material. The same ASV table used for Gneiss analysis was used to compile a list of ASVs present in each sample group. Once lists of ASVs were compiled for the transplant and sample groups, intersections between sets of ASVs were visualized using UpSet (59). ASVs which were present only in the transplant were removed to facilitate visualization of other intersections. Based on their presence in intersections, ASVs were classified as successfully transplanted, possibly transplanted, or environmental. ASVs were classified as successfully transplanted if they were present in the transplant and in treated chicks at least one time point before control chicks. ASVs were classified as possibly transplanted if they were present in the transplant and in both treated and control chicks at the same time point. ASVs were classified as environmental if they were present in the transplant and present in only control chicks or present in control chicks before treated chicks. Any ASV not present in the transplant was classified as environmental.

A chi-square test of independence was performed to examine the relationship between ASVs identified as differentially abundant between treatment groups and those defined as successfully transplanted, possibly transplanted, or environmental using Python’s SciPy module. The taxonomy of ASVs classified as successfully transplanted, possibly transplanted, and environmental was compared to that of ASVs identified as more abundant in treated chicks, more abundant in control chicks, and not differentially abundant with a hybrid Sankey diagram created using Sankeyview version 1.7.7 (60).

### Quantitative PCR.

Taxa were selected for further testing using quantitative PCR based on results from Gneiss analysis. A literature search was conducted to find suitable primers. Where suitable primers were not available, the sequences retrieved from Illumina sequencing were used to produce taxa-specific primers. The sequence was input into Primer-BLAST, and a suitable primer pair was chosen. To test the specificity of primers, each primer pair was input into TestPrime for comparison against the SILVA database SSU-r132. Further testing of primers was conducted using PCR. The primers were tested against known positive and negative samples to check for the correct amplicon size and nonspecific amplification. A gradient PCR was conducted to establish the correct annealing temperature for quantitative PCR. The primers used are displayed in Table 5.

**TABLE 5:** Primer pairs used for quantitative PCR

Target taxa	Primers^*a*^	Amplicon size (bp)	Reference
Domain *Bacteria* (targets V4 region)	F: TGCCAGCMGCCGCGGTAA	254	49
	R: GGACTACHVGGGTWTCTAAT		
*Clostridium*	F: TGCCAGCMGCCGCGGTAA	131	65
	R: GGACTACHVGGGTWTCTAAT		
*Enterobacteriaceae*	F: GTGCCAGCMGCCGCGGTAA	429	66
	R: GCCTCAAGGGCACAACCTCCAAG		
“Candidatus *Savagella*”	F: GATGCGTAGGCGGTTGAGTA	90	This study
	R: GGGTTTCTAATCCTGTTTGCTCC		
*Clostridium* cluster IV	F: TTACTGGGTGTAAAGGG	580	67
	R: TAGAGTGCTCTTGCGTA		
*Clostridium* cluster XIV (a and b)	F: AAATGACGGTACCTGACTAA	438–441	68
	R: CTTTGAGTTTCATTCTTGCGAA		

a F, forward; R, reverse.

The real-time quantitative PCR assay was conducted on a 1:10 solution of extracted DNA using a Rotor-Gene Q PCR machine (Qiagen) and PrecisionPLUS qPCR master mix (Primer Design, UK). The V4 region of the 16S rRNA gene was used as a reference gene. Rotor-Gene Q software version 2.3.1.49 was used to produce melting curves and identify the cycle threshold (*C_T_*), the point at which fluorescence above the background level is detectable. Each sample was run in triplicate with an averaged *C_T_* used in further analysis. The Δ*C_T_*, defined as the difference between the *C_T_* value for taxon-specific primers and the *C_T_* value for the reference gene, was calculated for each sample. Results were expressed as 40 – Δ*C_T_*. Amplification of DNA in one PC3 sample failed in all reactions. As a result, this sample was excluded from quantitative PCR analysis.

### Hematoxylin and eosin staining.

Tissue fixed in 4% paraformaldehyde solution was examined histologically to identify differences in morphological development of the ileum and cecal tonsil between treated and control chicks. Four sections of ileum and four sections of cecal tonsil from each chick underwent tissue processing using a Tissue-Tek vacuum infiltration processor (VIP) overnight before being embedded in paraffin (Ultraplast premium embedding medium, Solmedia). Paraffin sections of 4 μm were cut on a Leica RM2125 RT microtome, floated on a water bath, and placed on color slides (Solmedia, MSS54511YW). For hematoxylin and eosin (H&E) staining, slides were dewaxed in xylene and rehydrated through descending grades of ethanol (100%, 96%, 85%, 70%) to distilled water before being stained in hematoxylin (5 min), “blued” in tap water for 5 min, and stained in eosin (2 min). Slides were then dehydrated through 96% and 100% ethanol to xylene and cover-slipped using dansyl-polymyxin (DPX) (Thermo Scientific, Lamb/DPX). Hematoxylin (Atom Scientific, RRBD61-X) and eosin (TCS, HS250) solutions were made in-house.

Hematoxylin and eosin-stained tissue sections were examined using light microscopy (Nikon Eclipse 80i) with a Leica DMC 4500 digital camera attachment (Leica Microsystems, Switzerland). Images were viewed and measurements taken using Leica Application Suite X software.

Sections were assessed for suitability based on orientation of tissue samples. Villus height, villus width, and epithelial cell height were recorded in transverse ileal sections, where entire villi could be visualized to the lamina propria. In such sections, the height and width of five villi with an intact lamina propria were measured. Villus height was defined as the distance from the villus tip to the villus-crypt junction. Villus width was measured at the widest section of the villus. Epithelial cell height was measured at the villus tip and was defined as the distance from the distal point of the microvilli to the basement membrane. Measurements were expressed as a mean for each bird.

Mitotic figure counts in the ileum and cecal tonsil were used as an indication of intestinal villus development (61, 62). All orientations of tissue were included for mitotic counts where crypts were visible adjacent to the lamina propria and muscular layers. Mitotic figures in crypts within one high-power field (400×) of the lamina propria were counted. Cells were identified as mitotic if their nuclei were strongly basophilic and homogenous, with care taken to count cells in the late stages of division as a single mitotic figure. The length of lamina propria over which mitotic figures were counted was measured, and results were expressed as the number of mitotic figures per 100 μm. Results were expressed as a mean for each bird.

Results were compared between treatment groups using Student’s *t* test implemented in the SciPy version 1.1.0 Python module (63). A Benjamini-Hochberg false discovery rate correction implemented in the Statsmodels version 0.9.0 Python module (64) was applied to account for multiple tests. Results were compared between age groups using a one-way analysis of variance (ANOVA) test with a *post hoc* Tukey honestly significant difference (HSD) test when significant differences were identified.

During the analysis, it was noted that some samples had large aggregates of bacterial cells within the crypts of the cecal tonsil. In order to ascertain whether the presence of bacteria in the cecal tonsil crypts was associated with age or treatment group, slides were reexamined. Samples were classified as positive if bacteria were observed in more than one crypt and in at least two sections.

### Immunostaining.

Serial 7.5-μm-thick sections of cecal tonsil tissue frozen in OCT were cut using a cryostatic microtome. Four sections of cecal tonsil from each bird were mounted on poly-l-lysine-coated slides (VWR International, UK) and fixed in acetone for 10 min. Immunostaining was performed on a Dako Autostainer Link 48 using Envision FLEX reagents. Following a buffer rinse, tissue sections underwent a peroxidase block for 5 min (Agilent, SM801) before being incubated for 20 min with mouse monoclonal antibodies against chicken CD4, CD8α, CD8β, γδ TCR, and Bu1 (B cells and subsets of monocytes and macrophages) antigens (Cambridge Bioscience Ltd.; 8210-01, 8220-01, 8280-01, 8230-01, and 8395-01, respectively). The antibodies CD4 (1:200), CD8α (1:200), CD8β (1:1000), γδ TCR (1:100), and Bu1 (1:400) were diluted in Envision FLEX antibody diluent (Agilent, K8006). Antibody binding was detected using the labeled polymer Envision FLEX/HRP (Agilent, SM802) for 20 min, and the reaction was visualized using the substrate-chromogen FLEX DAB+Sub Chromo (Agilent, DM827 and SM802). Tissue sections were counterstained for 5 min in Envision FLEX hematoxylin (Agilent, K8008), washed in deionized water, and dehydrated through increasing grades of ethanol (85%, 96%, 3 × 100%) before clearing in xylene and mounted as for H&E staining above. All intermediate buffer washes between reagents used Envision FLEX wash buffer (K8007).

Stained tissue sections were examined using the same apparatus as described for hematoxylin and eosin-stained tissue. Quantification of cell abundance between treated and control chicks was performed by counting cells in photographs taken at a magnification of 200×, with each field of view representing an area of 142,000 μm^2^. Five photographs for each bird taken randomly from serial sections were used. Results were expressed as a mean for each bird. Student’s *t* test was used to identify significant differences in cell abundance between treatment groups.

### Data availability.

Raw sequence reads are available in the NCBI Sequence Repository Archive under BioProject PRJNA517619.

## Supplementary Material

Supplemental file 1
