# Supplementary material for: Topical Application of Adult Cecal Contents to Eggs Transplants Spore-Forming Microbiota but Not Other Members of the Microbiota to Chicks
Source: Appl Environ Microbiol. 2020 Feb 18;86(5):e02387-19. doi: 10.1128/AEM.02387-19 (PMC7028958; doi:10.1128/AEM.02387-19)

Table S1. Composition of starter and grower diets

Analytical Constituents (%)	Diet	
	Starter	Grower
Crude Fat	2.7	2.4
Crude Protein	18.9	15.6
Crude Fibre	3.8	4.1
Crude Ash	6.6	5.6
Lysine	0.99	0.69
Methionine	0.44	0.27
Calcium	1.05	0.89
Phosphorus	0.7	0.62
Sodium	0.15	0.15
Magnesium	0.17	0.22
Copper	15mg/kg	16 mg/kg

Figure S1 – Gel images showing PCR products from **A)** Standard dilutions of bacterial DNA from 10^6, 10^5, 10^4, 10^3, 10^2 and 10^1 bacterial cells including a positive (PC) and negative control (NC). **B)** DNA extracted from amniotic fluid from control (A) and treated (B) eggs; duodenum, jejunum and ileum from control (C) and treated (D) embryos and caeca from control (E) and treated (F) embryos including a positive and negative control. **C)** Samples spiked to include bacterial DNA along with a negative and positive control.

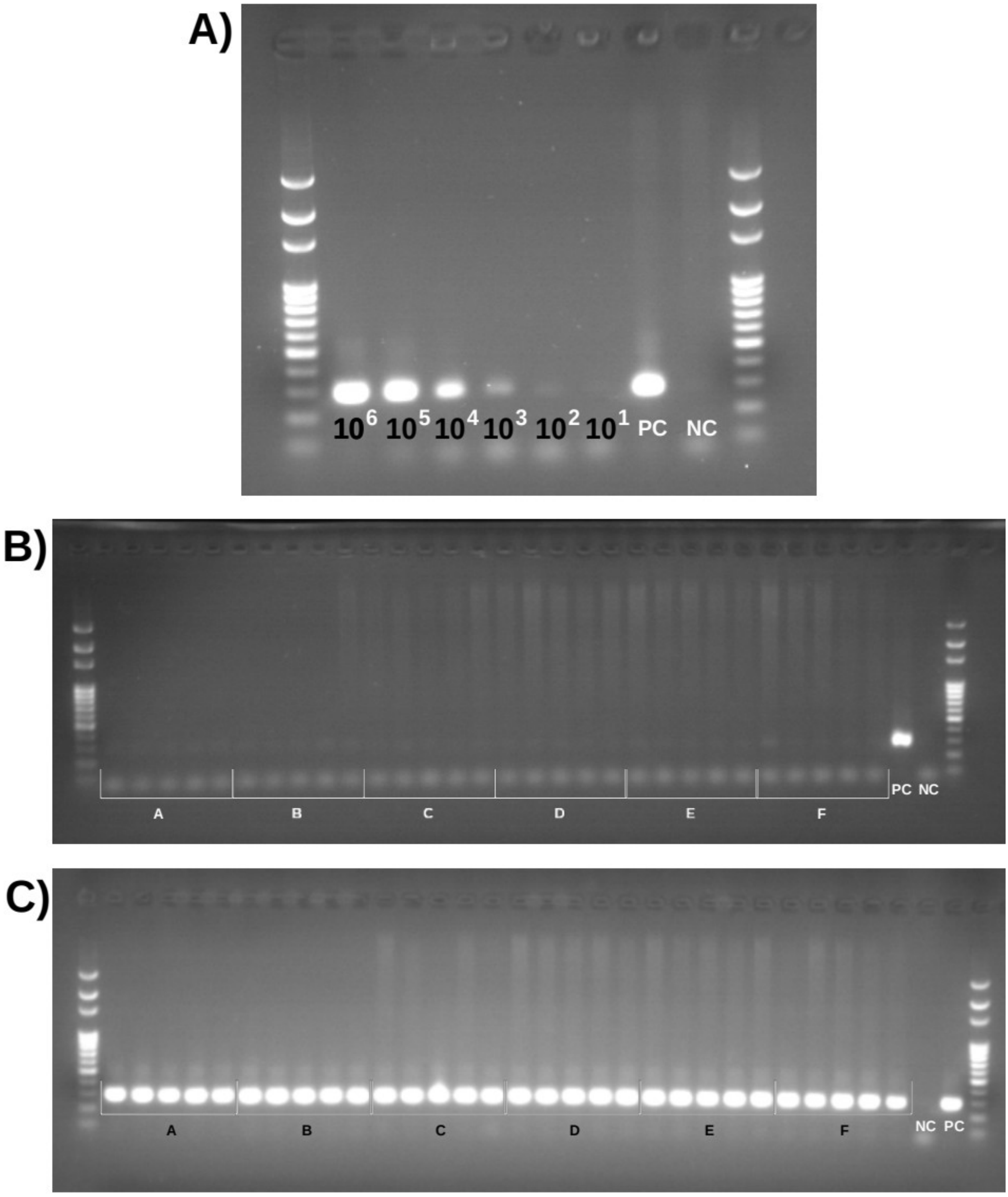

Figure S2 - A categorical heatmap comparing alpha diversity between sample groups. Alpha diversity between groups was compared using a pairwise Kruskal-Wallis test. Significant results between groups are indicated in red. Each box is annotated with the pairwise Kruskal-Wallis test statistic.

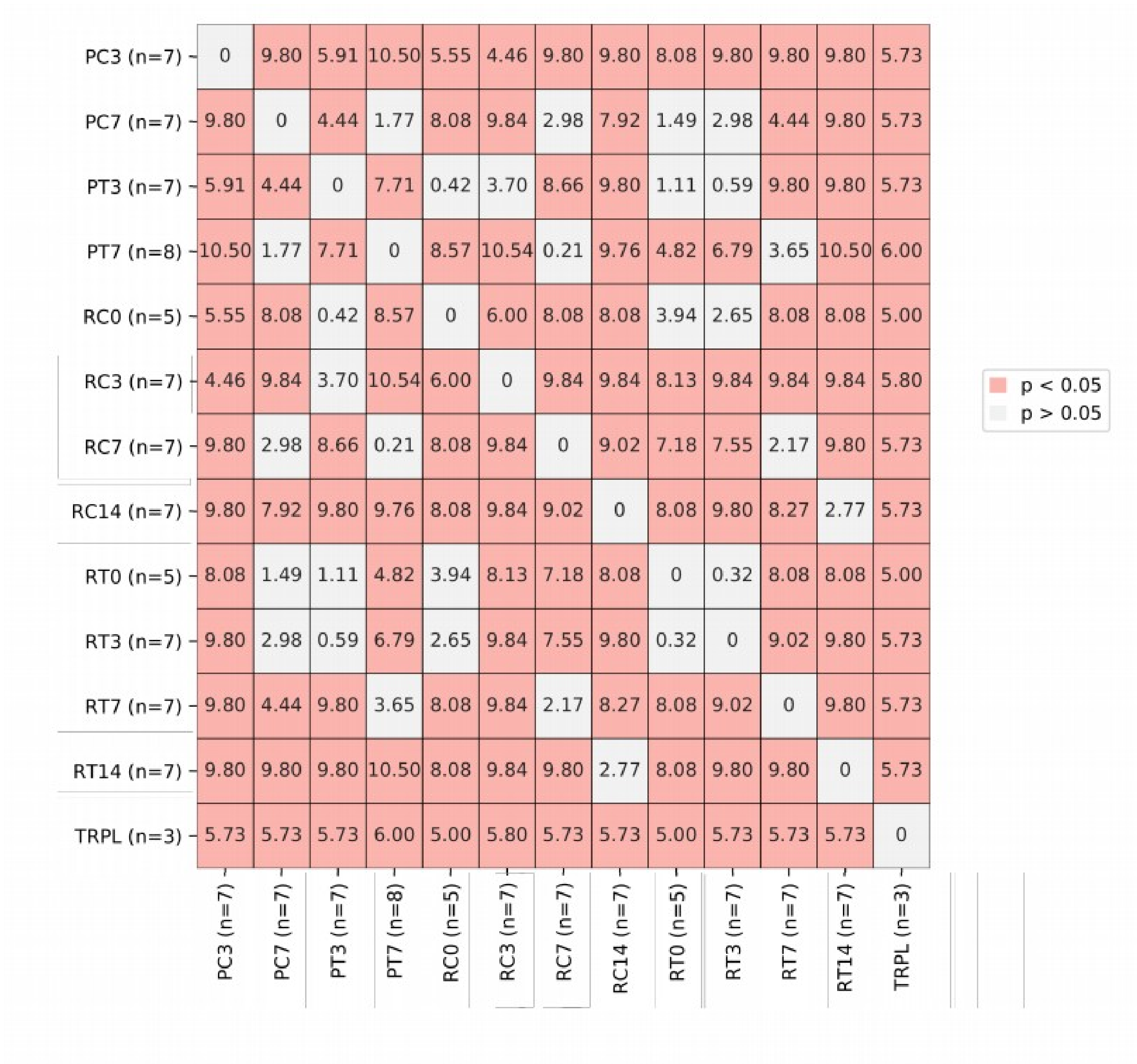

Figure S3 - A heatmap showing the average unweighted UniFrac distance between sample groups. Average unweighted UniFrac distances between sample groups was calculated to evaluate similarities in microbiome composition. A smaller distance between groups indicates increased similarity in microbiome composition. Treated samples were closer to TRPL samples than control samples of a similar age across both experiments with the effect most noticeable at 3 and 7 d.p.h.

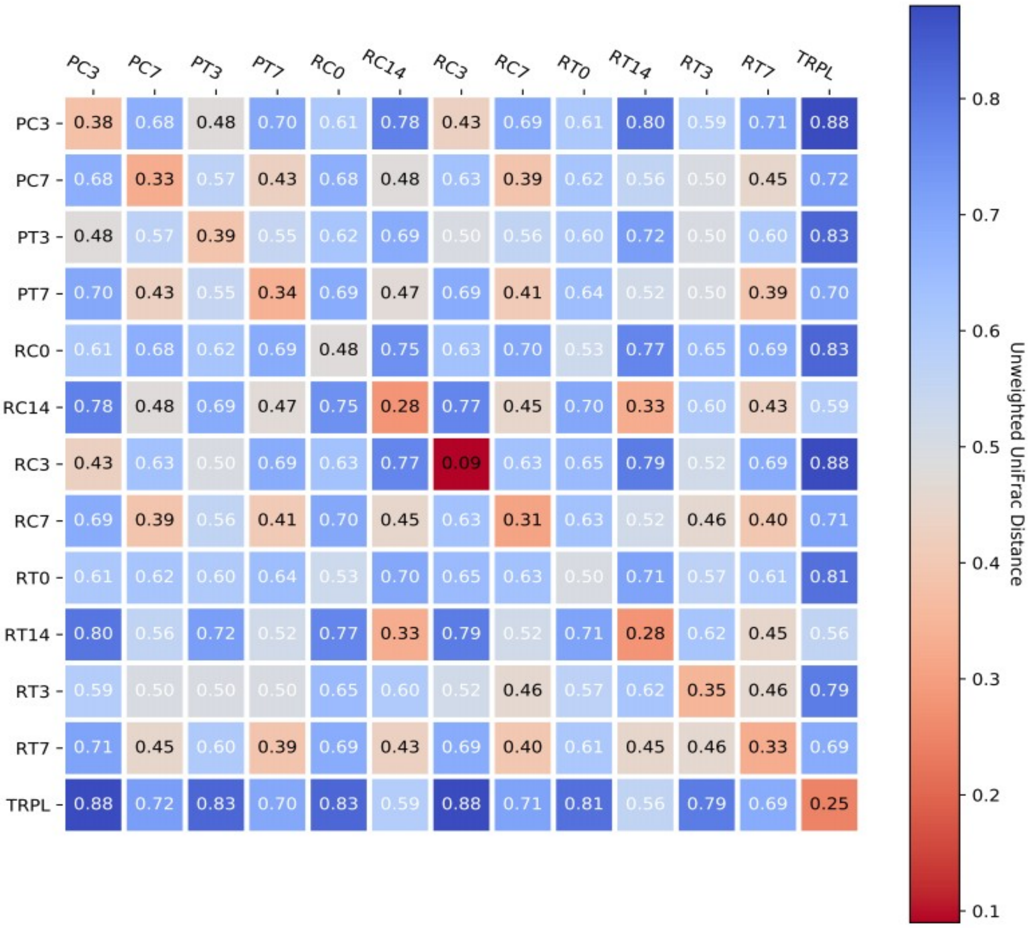

Figure S4 – Log ratios of balances y0 **(A)**, y2 **(B)**, y5 **(C)**, y6 **(D)**, y14 **(E)** and y27 **(F)** which were significantly different between treated and control chicks in the pilot experiment. A lower log ratio value suggests a shift in the balance towards denominator taxa either due to decreased abundance of numerator taxa or increased abundance of denominator taxa. In conjunction with the dendrogram heatmap (Figure 3) and taxa plot (Figure 4), ASVs which are differentially abundant between treated and control chicks were identified and displayed in Table 1.

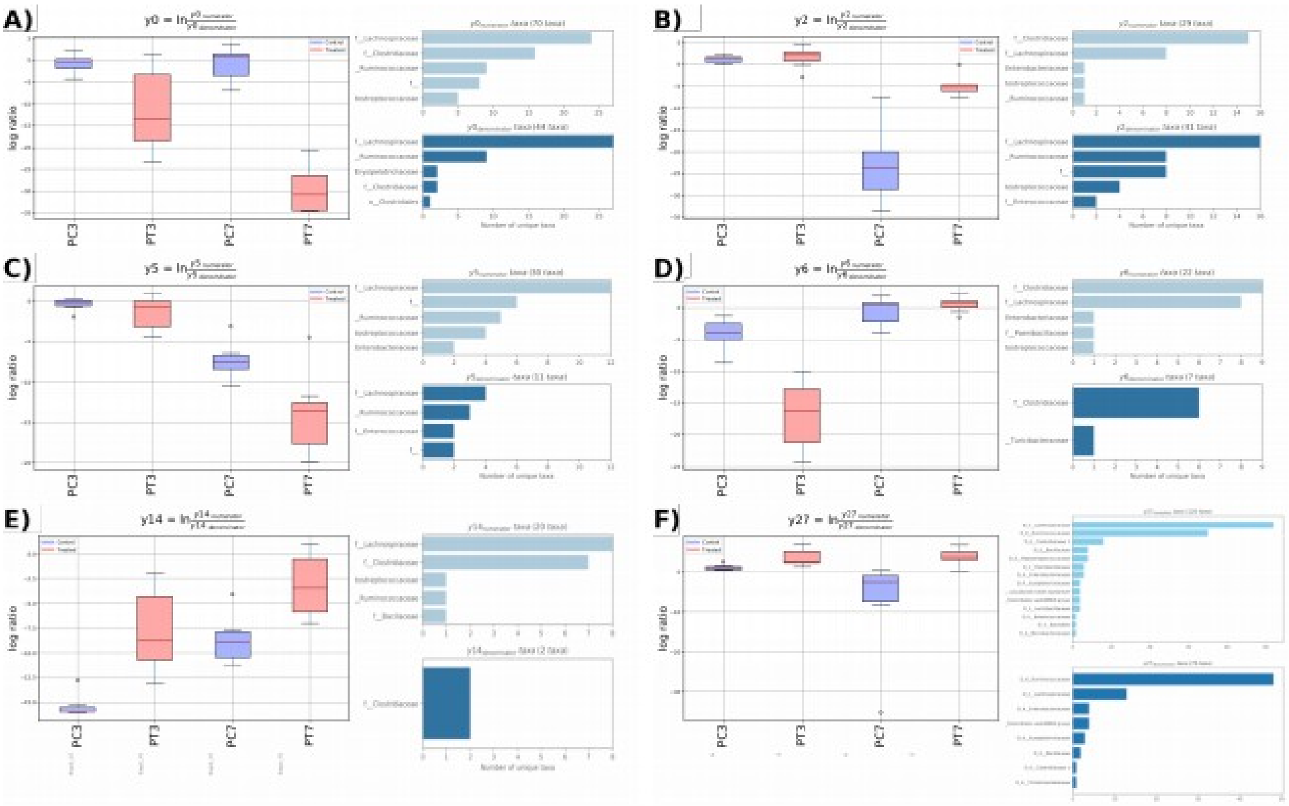

Figure S5 - The relative abundance of bacterial families in individual samples from each sample group. Early samples tended to be composed mainly of Clostridiaceae and Paenibacillaceae. These were replaced over time by *Lachnospiraceae* followed by *Ruminococcaceae* and order Clostridiales. These taxa tended to be found in treated samples at earlier time points than control samples with large differences in microbiota composition visible between samples from PC3 or RC3 and PT3 or RT3. These differences became less over time until little difference in the relative abundance of bacterial families is visible at 14 d.p.h.

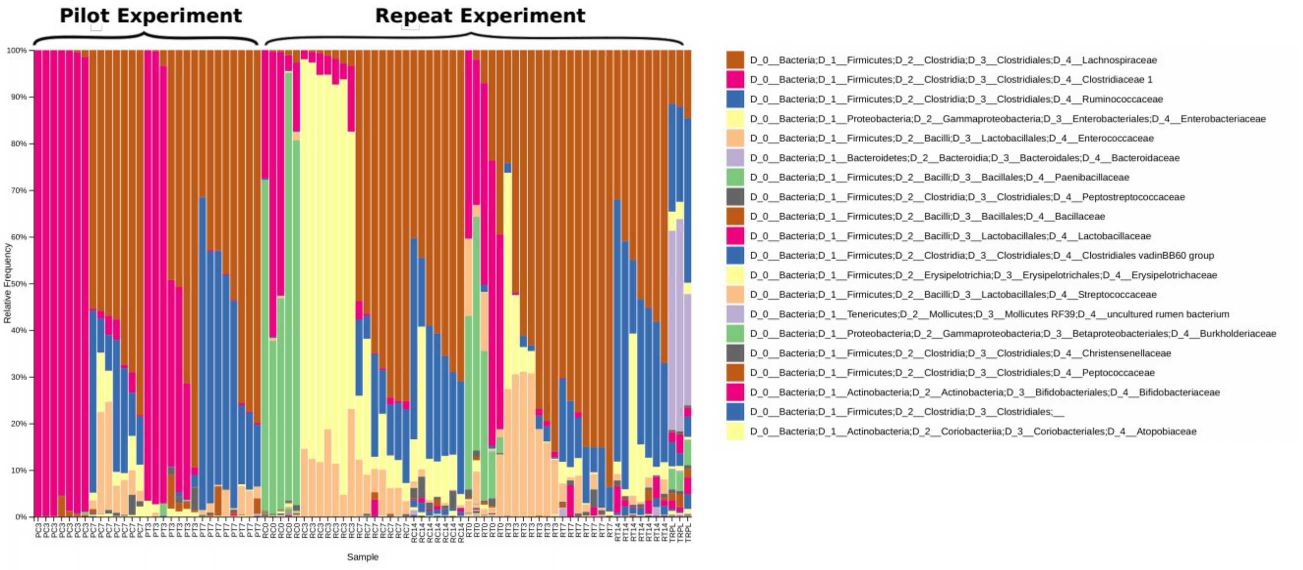

Figure S6 – Log ratios of balances y0 **(A)**, y4 **(B)** and y10 **(C)** which were significantly different between treated and control chicks in the repeat experiment. A lower log ratio value suggests a shift in the balance towards denominator taxa either due to decreased abundance of numerator taxa or increased abundance of denominator taxa. In conjunction with the dendrogram heatmap (Figure 5) and taxa plot (Figure 4), ASVs which are differentially abundant between treated and control chicks were identified and displayed in Table 2.

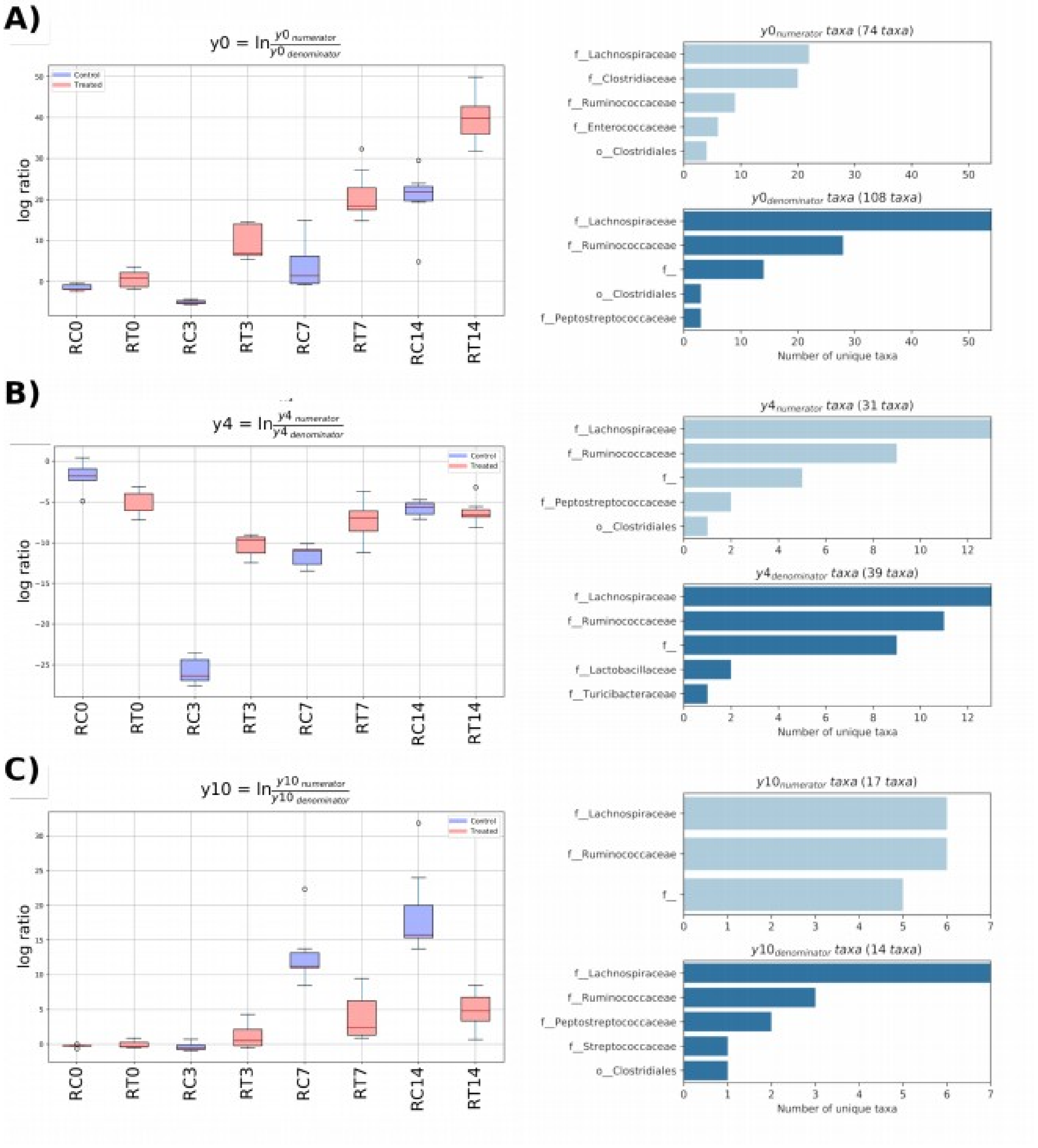

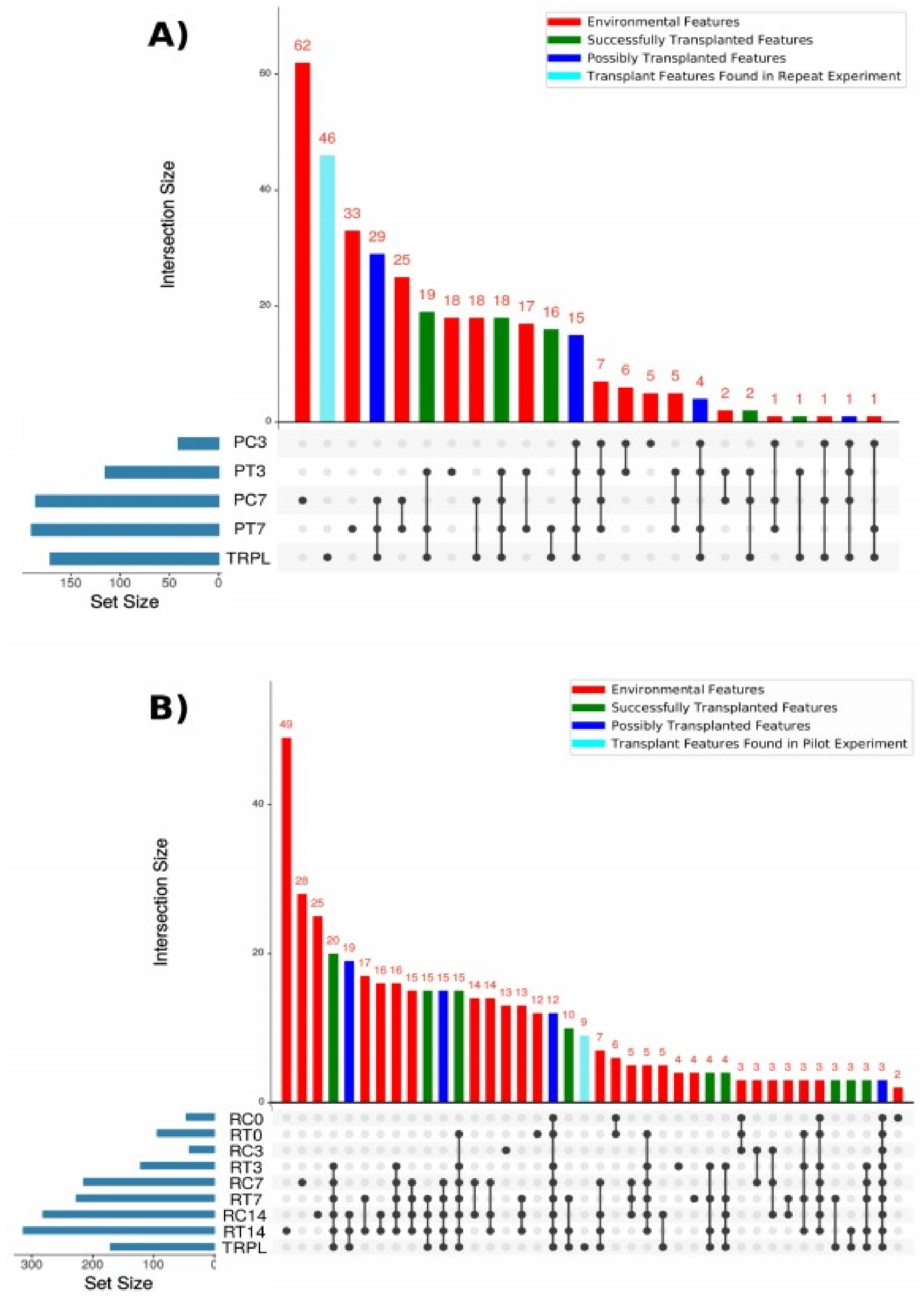

Figure S7 - Charts generated by UpSet showing the number of shared ASVs between sample groups in the pilot experiment **(A)** and repeat experiment **(B)**. Intersections are ordered by size with empty intersections excluded from the visualisation. The bar chart indicates the size of the intersection and is coloured by whether ASVs forming the intersection were classified as environmental, successfully transplanted or possibly transplanted. Intersections are visualised below the bar chart with contributing sample groups represented by black.

Figure S8 - Modified Sankey plots showing the relationship between ASVs identified as differential abundant in treated (TF), control (CF) and not differentially abundant (NF) and whether ASVs were classfied as successfully transplanted (ST), possibly transplanted (PT) or environmental (EV) in the pilot **(A)** and repeat **(B)** experiments. In both experiments, the majority of ASVs identified as differentially abundant in treated samples were also classified as successfully or possibly transplanted leading to the conclusion that differences between treated and control samples can be attributed to transplated ASVs rather than environmental ASVs.

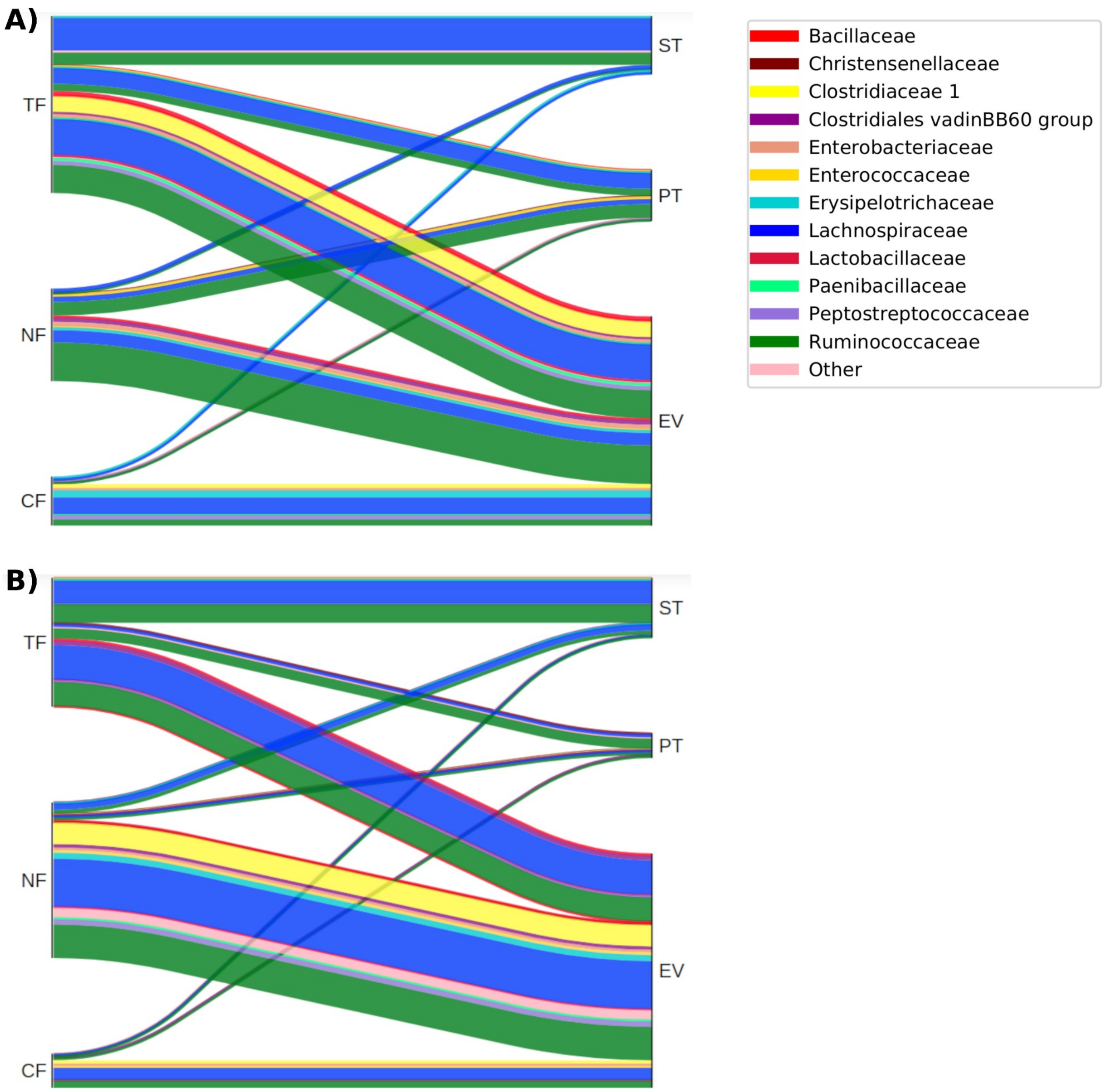

Figure S9 - Box and whisker plots showing mitotic figure counts in the ileum **(A)** and caecal tonsil **(B)**, epithelial cell height **(C)**, villus height **(D)** and villus width **(E)**. * $p < 0.05$.

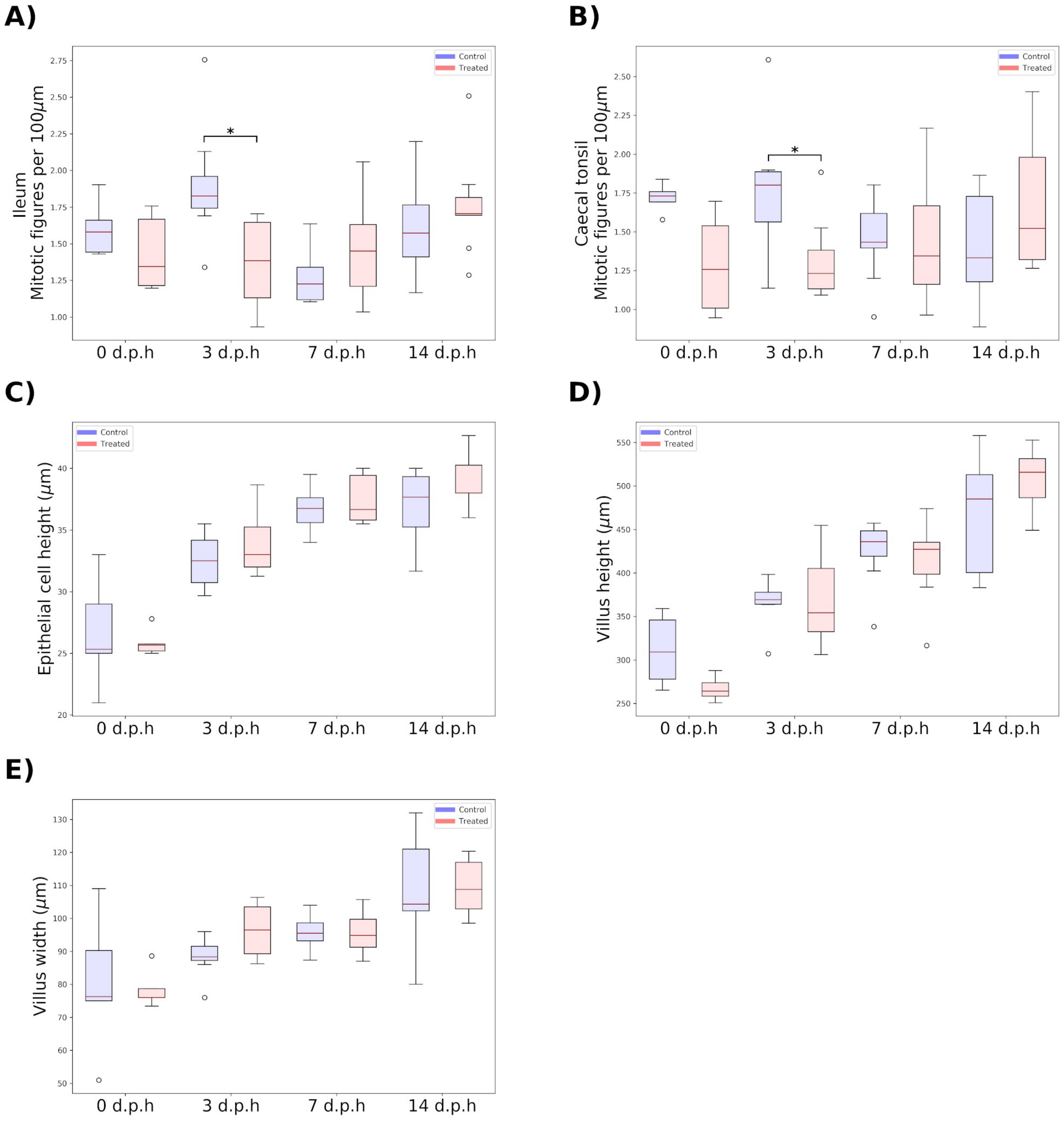

Figure S10 - Photographs showing close approximation of segmented filamentous bacteria to the caecal tonsil epithelium at 14 d.p.h **(A)** and presence of bacteria in the caecal tonsil crypts at 7 d.p.h **(B)**. Scale bar = 50μm, magnification: x 200.

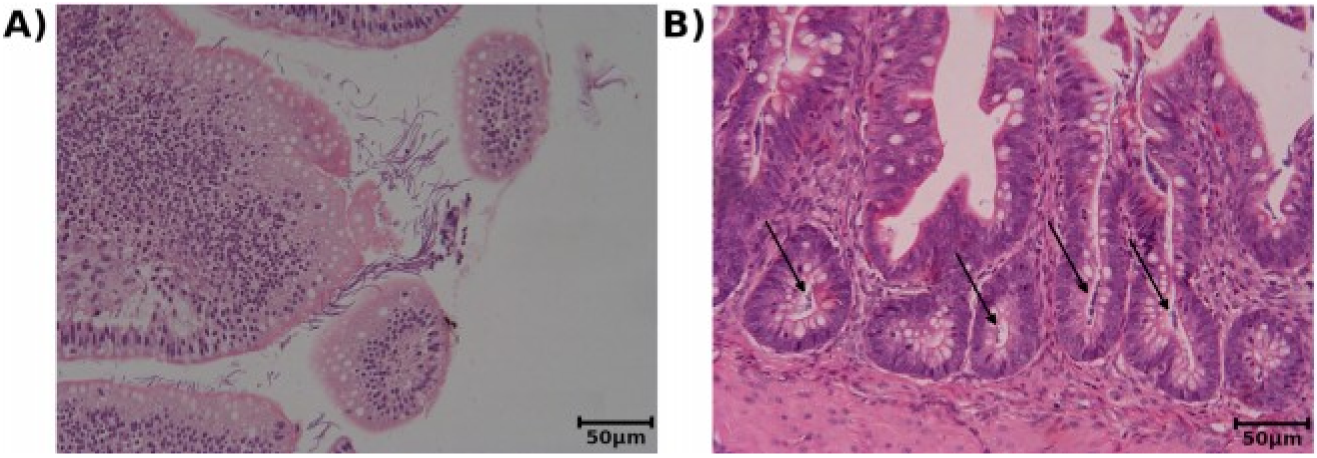

Supplement: Supplemental file 1 [file AEM.02387-19-s0001.pdf]
